# Glioblastoma cells that evade chemoradiotherapy-induced cell death exhibit a bifurcated glycolytic program

**DOI:** 10.1038/s41419-026-08646-9

**Published:** 2026-03-25

**Authors:** Emma Martell, Helgi Kuzmychova, Ujala Chawla, Akaljot Grewal, Charul Jain, Chitra Venugopal, Christopher M. Anderson, Sheila K. Singh, Tanveer Sharif

**Affiliations:** 1 Department of Pathology, Rady Faculty of Health Sciences, University of Manitoba, Winnipeg, MB Canada; 2 Department of Pharmacology and Therapeutics, Rady Faculty of Health Sciences, University of Manitoba, Winnipeg, MB Canada; 3 Pathology and Laboratory Medicine, Rady Faculty of Health Sciences, University of Manitoba, Winnipeg, MB Canada; 4 Department of Surgery, McMaster University, Hamilton, ON Canada; 5 Centre for Discovery in Cancer Research, McMaster University, Hamilton, ON Canada; 6 PrairieNeuro Research Centre, Health Sciences Centre, Kleysen Institute for Advanced Medicine, Winnipeg, MB Canada; 7 Department of Biochemistry, McMaster University, Hamilton, ON Canada; 8 Paul Albrechtsen Research Institute, CancerCare Manitoba, Winnipeg, MB Canada

**Keywords:** Cancer metabolism, CNS cancer, Cancer models

## Abstract

Glioblastoma (GBM), the most common malignant brain tumor in adults, remains a highly lethal and incurable cancer, with a 5-year survival rate below 10%. Standard-of-care involves surgical resection followed by concurrent temozolomide chemotherapy and radiation treatment. While these interventions can effectively shrink tumors, they fail to eradicate all malignant cells. Small populations of GBM cells invariably survive and seed recurrent disease, leading to near-universal relapse and the formation of fatal recurrent tumors, typically within 1–2 years of treatment. Here, we investigated the metabolic features that define these surviving cell populations using ten patient-derived GBM models and matched orthotopic xenograft models exposed to a clinically relevant chemoradiotherapy regimen. By sampling living cells at defined treatment intervals and integrating ^13^C-glucose tracing, quantitative untargeted metabolomics, and nCounter metabolic gene expression profiling, we reconstructed the temporal evolution of glucose metabolism from therapy-naïve to post-treatment states. Across all models, GBM cells that evaded therapy-induced death exhibited a conserved and coordinated reorganization of glycolytic flux. These cells showed enhanced glucose uptake and elevated abundance of upper glycolytic enzymes such as HK1, while lower glycolytic enzymes, including ALDOA, GAPDH, ENO1, and LDHA, were suppressed, resulting in reduced lactate output. This bifurcation of glycolytic metabolism redirected carbon flux toward the pentose phosphate pathway and nucleotide biosynthesis, as well as mitochondrial metabolism, supported by the increased abundance of tricarboxylic acid cycle enzymes. Notably, these adaptations were conserved in recurrent patient-derived orthotopic xenograft tumors in vivo. Together, these findings reveal a fundamental and conserved metabolic state that defines GBM cells surviving chemoradiotherapy. This study deciphers a core metabolic architecture that enables tumor cell survival, persistence, and recurrence following therapy by shifting glycolytic flux away from lactate production to balance biosynthetic demands with mitochondrial metabolism.

## Introduction

Therapy resistance and tumor recurrence remain major barriers to achieving durable responses in cancer treatment. This challenge is particularly evident in glioblastoma (GBM), an aggressive adult brain tumor with a 5-year survival rate below 10% [1]. Despite maximal surgical resection followed by concurrent chemotherapy and radiation, most patients experience relapse within 7–9 months [2–4]. Recurrent tumors are highly resistant to subsequent therapy, leading to near-universal progression and a median survival of only 12–18 months [4, 5].

Inter- and intra-tumoral heterogeneity is a defining feature of GBM [6–14]. While standard therapies may be effective at targeting and killing the bulk of tumor cells, the extensive cellular diversity and plasticity of GBM tumors almost guarantee the survival of small subpopulations with unique biological properties capable of adapting, persisting, and driving tumor regrowth [10, 14]. Characterizing the conserved molecular characteristics that enable these surviving populations to persist across genetically diverse tumors is crucial for understanding the biology of recurrent GBM and informing the design of more effective therapeutic strategies.

Metabolic networks function as ancient stress-protection mechanisms that can operate independently of transcriptional changes to establish an initial defense against external pressures [15]. Our prior work demonstrated that GBM cells exposed to chemoradiotherapy develop a dependence on the mitochondrial pyruvate carrier (MPC) to sustain mitochondrial reactions that regulate the abundance of substrates used for epigenetic modifications [16]. This process suppresses differentiation programs, maintaining a stem-like, therapy-resistant state that promotes recurrence [16]. While these findings uncovered a key mechanism linking mitochondrial metabolism and epigenetic control, it remains unclear whether chemoradiotherapy-surviving GBM cells undergo broader, coordinated metabolic reprogramming.

Glucose metabolism is of particular interest given that the brain consumes roughly 20% of total systemic glucose, despite making up only 2% of our body mass [17]. Within the cell, glucose is primarily catabolized through the glycolysis pathway, which not only supports ATP generation but also provides key intermediates for anabolic biosynthesis. Enhanced glycolytic flux, a hallmark of cancer known as the Warburg effect [18, 19], is prominent in GBM [20–23]. We have previously shown that stem-like cell populations from therapy-naïve patient tumors display elevated glycolysis and metabolic plasticity relative to their non-stem-like counterparts [24]. However, the temporal dynamics of glycolytic activity during chemoradiotherapy treatment and recurrent GBM progression remain poorly understood. Addressing this knowledge gap is not only of therapeutic relevance, given ongoing trials targeting glycolytic pathways in recurrent GBM [25–27], but also of fundamental biological importance for understanding how tumor cells dynamically modulate glucose utilization under stress.

The stress response induced by chemoradiotherapy is inherently dynamic, and the transient metabolic states that arise during treatment may not persist once tumors relapse. Consequently, comparisons between primary and recurrent tumor samples offer only a static end-state snapshot. Furthermore, the scarcity of fresh recurrent patient samples limits the feasibility of in-depth metabolic profiling, as many assays require viable tissue rather than fixed archived material. To overcome these challenges, we employed clinically relevant in vitro and in vivo patient-derived GBM models that recapitulate the standard chemoradiotherapy regimen used in patients [16, 28, 29]. By longitudinally sampling living tumor cells at defined treatment timepoints, we reconstructed the temporal progression of metabolic states as GBM cells shift from therapy-naïve to post-treatment phenotypes.

Here, we reveal a universal metabolic feature occurring at the critical bottleneck point of chemoradiotherapy treatment, where cells either perish or persist to give rise to recurrent tumors. Across ten patient-derived models, therapy-surviving cells exhibit a bifurcated glycolytic phenotype characterized by enhanced glucose uptake and activation of upper glycolytic enzymes, with suppression of lower glycolysis and reduced lactate output. This coordinated glycolytic uncoupling reroutes upper glycolytic intermediates toward anabolic pathways, particularly those supporting nucleotide biosynthesis. Together, these findings reveal a fundamental and conserved metabolic signature that underlies GBM cell persistence following chemoradiotherapy, offering new insight into the core biological processes that drive tumor recurrence and disease progression.

## Results

### GBM cells that survive chemoradiotherapy treatment exhibit increased glucose uptake but decreased lactate production

**Fig. 1:**
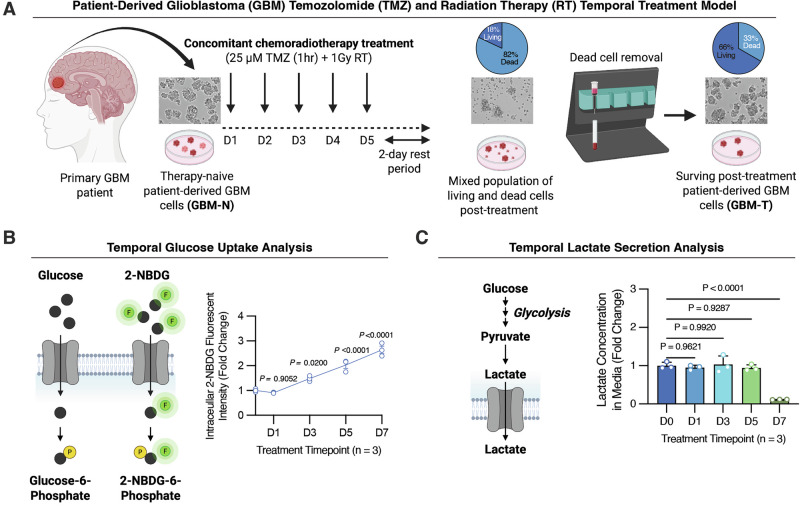
GBM cells that survive chemoradiotherapy treatment exhibit increased glucose uptake but decreased lactate production. **A** Schematic depicting a clinically relevant in vitro chemoradiotherapy treatment model of patient-derived GBM to study temporal molecular adaptation. BT935 patient-derived GBM cells treated with TMZRT were collected at D0 (no treatment therapy-naïve control), D1, D3, D5, and D7 time-points and living cells were isolated and subjected to: **B** 2-NBDG glucose uptake analysis and **C** extracellular lactate levels measurement. *N* = 3 biological replicates/group, mean ± SEM analyzed using one-way ANOVA test with Dunnett’s multiple comparisons.

GBM patients are treated with an aggressive combination of temozolomide (TMZ) and craniospinal radiation therapy (RT) [4]. While this treatment efficiently kills most tumor cells, a small subset may evade therapy-induced cell death and ultimately drive disease recurrence. To investigate the metabolic mechanisms underlying survival following therapy, we applied our previously validated and clinically relevant in vitro concurrent TMZRT treatment protocol (Fig. 1A) [16, 28]. GBM cells derived from primary patient tumors that had not previously been exposed to chemoradiotherapy, were treated daily with 25 µM TMZ for 1 h along with 1 Gy of concurrent RT for five consecutive days, followed by a two-day recovery period (Fig. 1A). Trypan blue exclusion revealed that while around 82% of GBM cells died, ~18% of cells survived this treatment (Fig. 1A). To enrich for this viable subpopulation, dead cells were removed using magnetic bead sorting, which eliminated roughly 60% of the dead cells and increased the proportion of living cells to nearly 70% (Fig. 1A). This model enables comparison between therapy-naïve GBM (GBM-N) and post-treatment surviving GBM (GBM-T) cells and permits temporal sampling of cells to capture dynamic metabolic phenotypes during the treatment course (Fig. 1A). Surviving cell populations were collected and analyzed at days 1 (D1), 3 (D3), 5 (D5), and 7 (D7) of treatment, alongside matched vehicle-treated controls (Fig. 1A).

Given the central role of glucose metabolism in GBM [20–23], we monitored glucose uptake using the fluorescent glucose analog 2-deoxy-2-[(7-nitro-2,1,3-benzoxadiazol-4-yl) amino]-D-glucose (2-NBDG) and glycolytic output by measuring extracellular lactate secretion in the culture medium (Fig. 1B, C) [30]. We observed a progressive and significant increase in glucose uptake across the treatment time points (Fig. 1B). Surprisingly, however, extracellular lactate levels remained relatively stable throughout the first 5 days of the treatment protocol but markedly declined in D7 GBM-T cells (Fig. 1C). These findings suggest a decoupling between glucose uptake and glycolytic flux following chemoradiotherapy.

### Temporal profiling of glycolytic enzymes reveals bifurcated regulation of upper and lower glycolysis

**Fig. 2:**
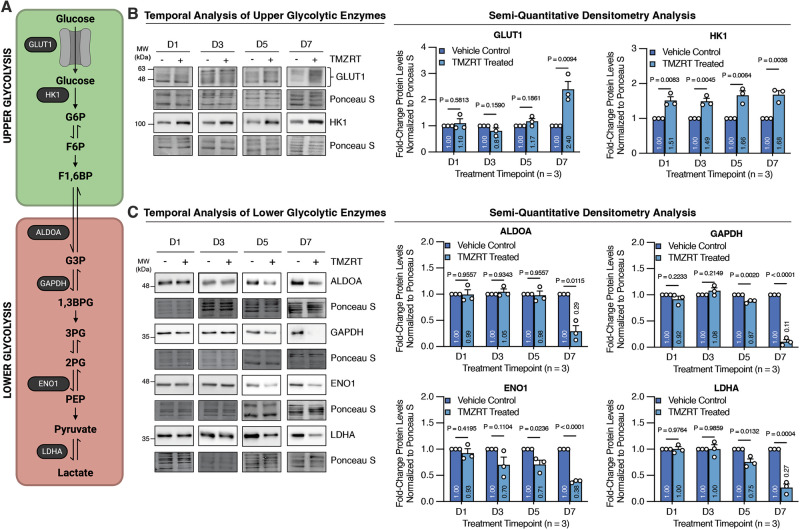
Temporal profiling of glycolytic enzymes reveals bifurcated regulation of upper and lower glycolysis. **A** Schematic depiction of glucose uptake, catabolism, metabolic intermediates, and the enzymes catalyzing the reactions of upper and lower glycolysis. BT935 patient-derived GBM cells treated with placebo control or TMZRT were collected at D1, D3, D5, and D7 time-points and living cells were isolated and subjected to: **B** immunoblot and semi-quantitative densitometry analysis of protein levels for upper glycolytic enzymes GLUT1 and HK1 and **C** lower glycolytic enzymes ALDOA, GAPDH, ENO1, and LDHA. *N* = 3 biological replicates/group, mean + SEM analyzed using unpaired two-tailed t-test.

Glycolysis can be conceptually divided into two phases: upper and lower glycolysis (Fig. 2A). In upper glycolysis, glucose is converted into 6-carbon intermediates: glucose-6-phosphate (G6P), fructose-6-phosphate (F6P), and fructose-1,6-bisphosphate (F1,6BP) (Fig. 2A). Whereas lower glycolysis processes these intermediates into 3-carbon metabolites: glyceraldehyde-3-phosphate (G3P), 1,3-bisphosphoglycerate (1,3BPG), 3-phosphoglycerate (3PG), 2-phosphoglycerate (2PG), phosphoenolpyruvate (PEP), and pyruvate, which can then be converted to lactate or oxidized in the mitochondria (Fig. 2A). To resolve the apparent paradox where we observed increased glucose uptake while glycolytic output declined in chemoradiotherapy-treated GBM cells, we performed a temporal analysis of key upper and lower glycolytic enzymes using immunoblotting, followed by semi-quantitative densitometric quantification of protein abundance normalized to total protein (Ponceau S) from three independent experiments (Fig. 2B, C). Total protein normalization was used instead of housekeeping controls, as conventional reference proteins such as glyceraldehyde-3-phosphate dehydrogenase (GAPDH), actin, or tubulin can vary significantly under metabolic stress, differentiation, or cell cycle changes [31–34].

We first examined the abundance of the main glucose transporter, GLUT1, and the first rate-limiting enzyme of upper glycolysis, hexokinase isoform 1 (HK1), which catalyzes the phosphorylation of glucose to G6P, so it remains trapped intracellularly (Fig. 2A). In agreement with the increased uptake and intracellular retention of 2-NBDG (Fig. 1B), HK1 expression rose sharply after the first day of treatment and remained elevated throughout the regimen, while GLUT1 expression robustly increased by D7 (Fig. 2B). In contrast, lower glycolytic enzymes such as aldolase A (ALDOA), GAPDH, enolase 1 (ENO1), and lactate dehydrogenase A (LDHA), were significantly reduced by D5 or D7 of treatment (Fig. 2A, C), which coincided with the observed reduction in extracellular lactate levels (Fig. 1C). Together, these findings point to a bifurcated glycolytic program that enhances glucose consumption while simultaneously reducing downstream glycolytic activity (Fig. 2C).

### Temporal stable isotope tracing reveals elevated glucose uptake and retention coupled with attenuated lower glycolytic flux and reduced glucose-derived lactate production in chemoradiotherapy-treated GBM cells

**Fig. 3:**
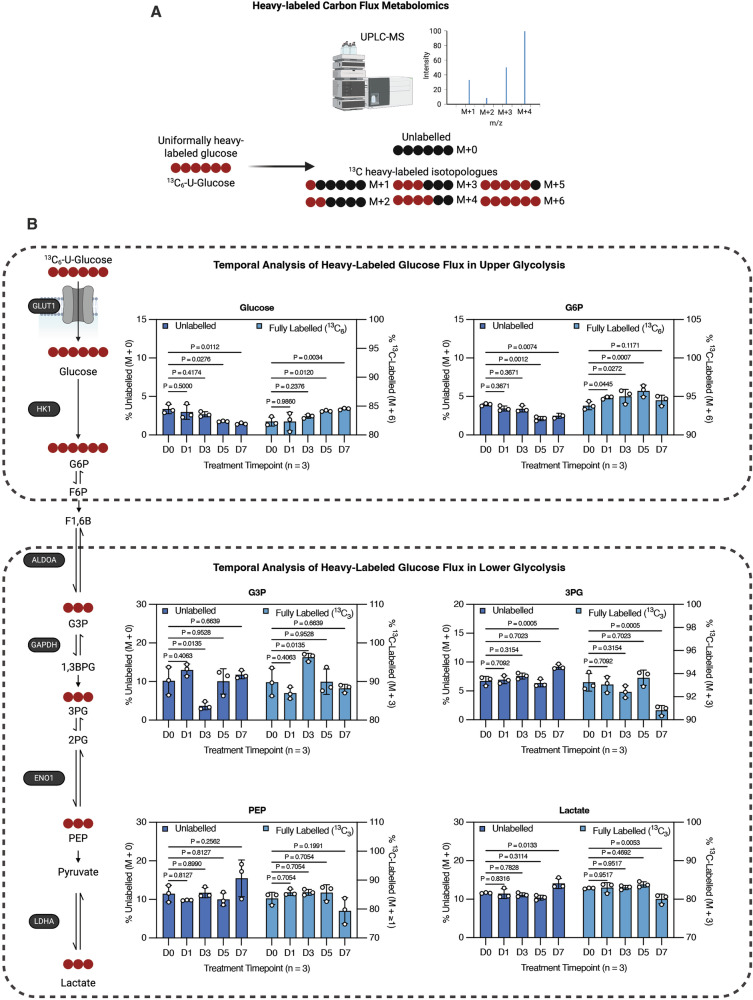
Temporal stable isotope tracing reveals elevated glucose uptake and retention coupled with attenuated lower glycolytic flux and reduced glucose-derived lactate production in chemoradiotherapy-treated GBM cells. **A** Schematic diagram depicting heavy-labeled carbon flux metabolomics analysis by mass spectrometry and heavy-labeled isotopologue patterns. **B** BT935 patient-derived GBM cells treated with TMZRT were collected at D0 (no treatment therapy-naïve control), D1, D3, D5, and D7 time-points and living cells were isolated and incubated with ^13^C_6_-U-glucose followed by mass spectrometry analysis for central carbon metabolites. Bar graphs represent the % of unlabeled (M + 0; left y-axis) and fully labeled (M + 3 or M + 6; right y-axis) of glucose, G6P, G3P, 3PG, PEP, and lactate. *N* = 3 biological replicates/group, mean ± SEM analyzed using two-way ANOVA test with Holm-Šídák’s multiple comparisons.

To more precisely monitor glucose metabolism, we performed stable isotope tracing in untreated D0 GBM-N cells and chemoradiotherapy-treated GBM cells collected at progressive time points (D1, D3, D5, and D7), incubated with uniformly heavy-labeled glucose (¹³C₆-U-glucose). Intracellular metabolites were extracted from living cells and analyzed by quantitative mass spectrometry (MS) (Fig. 3A). This approach enables direct quantification of glucose catabolism by tracking the incorporation of ¹³C heavy carbon atoms into downstream metabolites (Fig. 3A). Incorporation of ¹³C increases the molecular mass of metabolites by one unit per labeled carbon, allowing discrimination between unlabeled (M + 0) and partially or fully labeled isotopologues (M + 1, M + 2, … M + n) (Fig. 3A). Consistent with our 2-NBDG uptake data (Fig. 1B), intracellular ¹³C-glucose levels and fractional enrichment of fully labeled G6P (M + 6) significantly increased in chemoradiotherapy-treated GBM cells, confirming enhanced glucose uptake and retention (Fig. 3B and Supplementary Table 1). In contrast, ¹³C incorporation into intermediates of lower glycolysis exhibited a general decreasing trend over the treatment course (Fig. 3B and Supplementary Table 1). Although we observed a transient increase in labeled G3P at D3 of treatment (Fig. 3B), this is likely a consequence of early increases in upper glycolytic flux resulting in a temporary accumulation of labeled G3P, which resolves as ALDOA and lower glycolytic capacity declines at later treatment time points (Figs. 3B and 2C).

Notably, ¹³C-labeled lactate was significantly reduced in D7 GBM-T cells relative to untreated D0 GBM-N controls, although not as drastically as the depletion of total extracellular lactate levels, suggesting that lactate export may also be impaired (Figs. 3B and 1C**;** Supplementary Table 1). Indeed, analysis of our previously published nCounter gene expression dataset encompassing a panel of 768 metabolism-related genes [16] revealed that GBM cells surviving chemoradiotherapy have significantly reduced expression of the lactate monocarboxylate transporters (MCTs), MCT1 (*SLC16A1*) and MCT4 (*SLC16A3*) (Supplementary Fig. 1A, B). Together, these data corroborate our earlier biochemical findings, demonstrating that while chemoradiotherapy-treated GBM cells continue to avidly import and phosphorylate glucose, they progressively attenuate its downstream catabolism through lower glycolysis, suggesting a rerouting of glucose metabolism toward alternative metabolic fates.

### Bifurcated upper and lower glycolysis in chemoradiotherapy-surviving GBM cells is a conserved phenotype across diverse patient-derived models

**Fig. 4:**
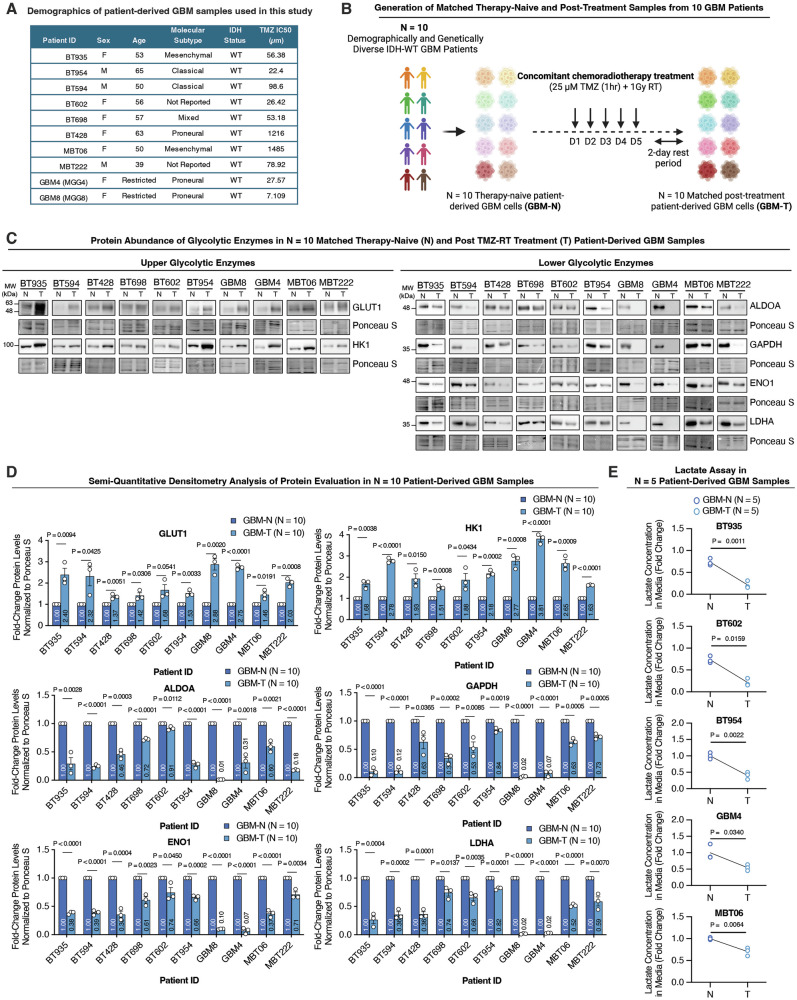
Bifurcated upper and lower glycolysis in chemoradiotherapy-surviving GBM cells is a conserved phenotype across diverse patient-derived models. **A** Table of demographics for 10 patient-derived GBM samples used in this study. **B** Schematic depicting a clinically relevant in vitro chemoradiotherapy treatment model used to generate matched therapy-naïve (N) and post-treatment (T) samples from 10 patients. *N* = 10 matched N, and T patient-derived GBM cells were subjected to **C** immunoblot analysis and **D** semi-quantitative densitometry analysis of protein levels for GLUT1, HK1, ALDOA, GAPDH, ENO1, and LDHA with *N* = 3 biological replicates per sample, where graphs represent mean + SEM, analyzed using an unpaired two-tailed t-test. **E** Extracellular lactate was measured in *N* = 5 matched therapy-naïve and post-treatment patient-derived GBM cells with *N* = 3 biological replicates per sample, analyzed using an unpaired two-tailed t-test.

GBM is well recognized for its robust inter-patient heterogeneity [6–14]. To determine whether this metabolic phenotype is conserved in chemoradiotherapy-surviving GBM cells across the diverse GBM patient landscape, we conducted a comprehensive metabolic analysis of ten distinct patient-derived GBM samples. These samples encompassed a range of clinical and genomic backgrounds and exhibited variable intrinsic sensitivity to TMZ (Fig. 4A) [16].

Each patient-derived culture was subjected to the same TMZRT treatment regimen, after which the surviving GBM-T populations were isolated for detailed metabolic protein profiling and functional assays (Fig. 4B). Remarkably, despite their clinical and molecular variability, GBM-T cells from all ten patients displayed a consistent glycolytic signature, where the abundance of GLUT1 and HK1 was significantly increased relative to matched GBM-N controls, while lower glycolytic enzymes ALDOA, GAPDH, and ENO1 were downregulated (Fig. 4C, D). Furthermore, LDHA expression was markedly reduced in GBM-T cells (Fig. 4C, D), corresponding with a significant decrease in extracellular lactate secretion (Fig. 4E). These findings indicate that simultaneous elevation of upper glycolytic enzymes with suppression of lower glycolytic enzymes and output is a conserved metabolic phenotype across heterogeneous GBM cells that survive chemoradiotherapy.

### Bifurcation of upper and lower glycolytic metabolism following chemoradiotherapy treatment is conserved in vivo and maintained in recurrent tumors

**Fig. 5:**
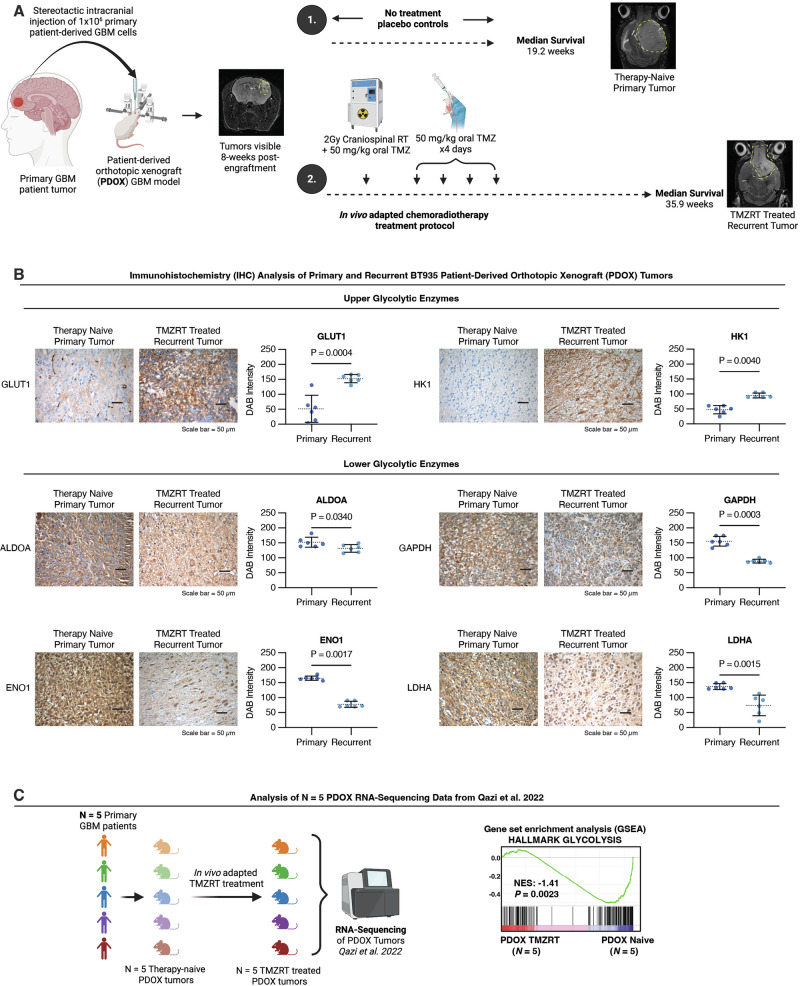
Bifurcation of upper and lower glycolytic metabolism following chemoradiotherapy treatment is conserved in vivo and maintained in recurrent tumors. **A** Schematic diagram of a clinically relevant in vivo protocol used to generate matched therapy naïve primary and TMZRT-treated recurrent PDOX BT935 tumors. MRI photos depict tumor progression at various stages of the treatment protocol. **B** Representative micrographs and quantifications of DAB intensity from IHC analysis for GLUT1, HK1, ALDOA, GAPDH, ENO1, and LDHA, in therapy naïve primary versus TMZRT-treated recurrent PDOX BT935 GBM tumor tissues. *N* = 6 samples/group analyzed using an unpaired two-tailed t-test. **C** GSEA of Hallmark Glycolysis enrichment in RNA-Seq data from TMZRT-treated versus therapy naïve in *N* = 5 PDOX GBM tumor models.

We next evaluated whether this distinct metabolic profile of chemoradiotherapy-surviving GBM cells observed in vitro is sustained in the recurrent tumors arising from these persisting cell populations in vivo. Using a patient-derived orthotopic xenograft (PDOX) model, BT935 GBM cells were implanted into the frontal lobe of NOD-SCID gamma (NSG) mice and tumor formation was confirmed by MRI at eight weeks post-engraftment (Fig. 5A). Animals were then randomized to receive either no treatment (therapy-naïve placebo controls) or a five-day concurrent chemoradiotherapy regimen optimized for the radiosensitive physiology of NSG mice, which we previously validated as a clinically relevant in vivo model of GBM treatment and recurrence (Fig. 5A) [16, 29]. Mice received 50 mg/kg of oral TMZ and 2 Gy of craniospinal RT on the first day, followed by four subsequent doses of 50 mg/kg TMZ daily (Fig. 5A). This chemoradiotherapy treatment regimen significantly prolonged the median survival of mice to 35.9 weeks compared to 19.2 weeks in mice without any treatment, but recurrent tumors eventually emerge (Fig. 5A) [16].

Immunohistochemistry (IHC) analysis comparing primary therapy-naïve and recurrent TMZRT-treated GBM xenografts revealed elevated GLUT1 and HK1 in recurrent tumors, coupled with reduced ALDOA, GAPDH, ENO1, and LDHA abundance (Fig. 5B). These findings were corroborated through gene set enrichment analysis (GSEA) of a previously published RNA-sequencing dataset from five distinct GBM PDOX models exposed to the same chemoradiotherapy treatment protocol as used in this study [29], where TMZRT-treated tumors exhibited decreased enrichment of hallmark glycolysis-related genes compared to therapy-naïve tumors (Fig. 5C). Together, these findings establish that the bifurcated glycolytic state of chemoradiotherapy-surviving GBM cells is a conserved and durable metabolic characteristic recapitulated in vivo and maintained through GBM recurrence.

### Reduction in lower glycolytic enzymes and lactate production corresponds with elevated abundance of mitochondrial metabolic enzymes in chemoradiotherapy-treated GBM

The decline in lower glycolytic flux and decreased lactate production from glucose-derived pyruvate align with our recent findings that chemoradiotherapy upregulates the MPC and increases the levels of glucose-derived ^13^C-labeling into multiple tricarboxylic acid (TCA) cycle metabolites, suggesting increased diversion of pyruvate toward mitochondrial metabolism [16]. To further validate this hypothesis, we analyzed the temporal protein expression of key mitochondrial TCA cycle enzymes, aconitase 2 (ACO2), succinate dehydrogenase A (SDHA), fumarate hydratase (FH), and malate dehydrogenase 2 (MDH2), during our in vitro chemoradiotherapy treatment course. All four enzymes were progressively upregulated throughout the regimen (Supplementary Fig. 2). This increase in TCA cycle enzyme abundance was consistently observed across all ten patient-derived GBM models (Supplementary Fig. 3) and in recurrent TMZRT-treated PDOX tumors compared to their therapy-naïve counterparts (Supplementary Fig. 4). Collectively, these data reveal a coordinated metabolic transition in which suppression of lower glycolysis coupled with enhancement of mitochondrial metabolism defines the bioenergetic landscape of therapy-surviving GBM cells.

### Chemoradiotherapy promotes nucleotide biosynthesis through metabolic rerouting of upper glucose flux toward the pentose phosphate pathway

**Fig. 6:**
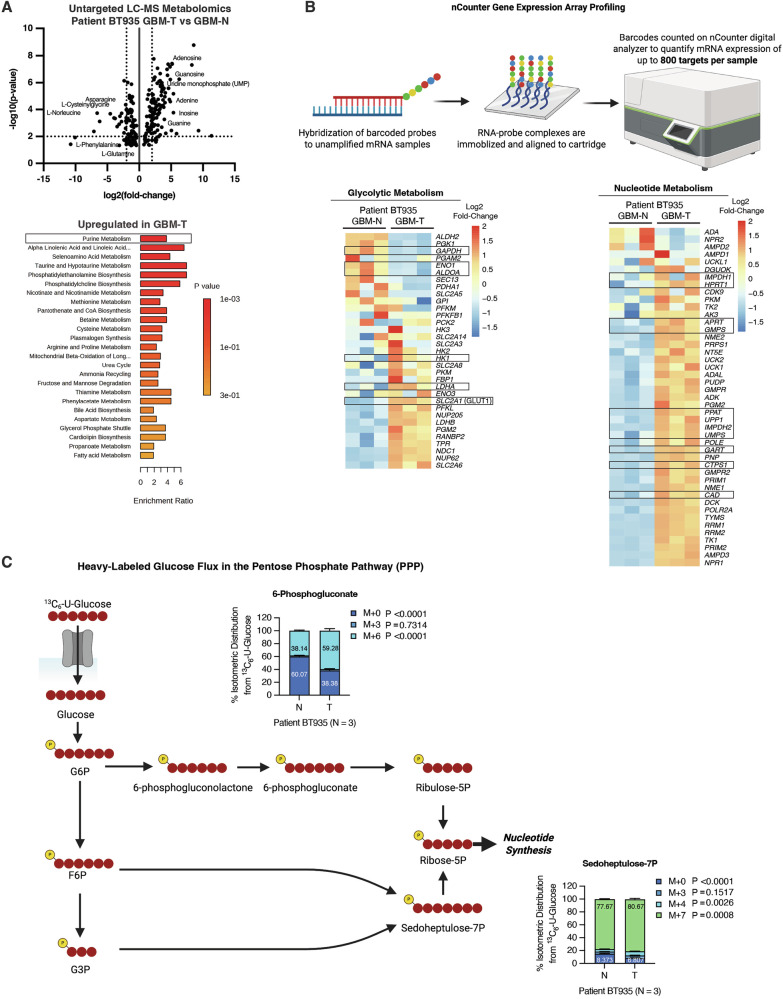
Chemoradiotherapy promotes nucleotide biosynthesis through metabolic rerouting of upper glucose flux toward the pentose phosphate pathway. **A** Therapy naïve (GBM-N) and post-treatment (GBM-T) patient-derived BT935 cells were subjected to untargeted global mass-spectrometry metabolomics analysis, and a volcano plot depicts fold changes in metabolites within GBM-T versus GBM-N cells, and a bar graph shows the functional enrichment of metabolic pathways upregulated in GBM-T versus GBM-N cells. **B** NanoString nCounter Metabolic Pathway panel analysis and heatmaps depicting log2 fold-change expression of glycolytic metabolism genes and nucleotide metabolism genes in GBM-T versus GBM-N cells from *N* = 3 biological replicates/group. **C** % ^13^C isotopologue abundance in PPP metabolites 6-phosphogluconate and sedoheptulose-7P and from ^13^C_6_-U-glucose in GBM-T versus GBM-N cells from *N* = 3 biological replicates/group.

To assess the broader metabolic consequences of glycolytic bifurcation, we performed global untargeted liquid chromatography-MS (LC-MS) metabolomics analysis on surviving GBM-T cells and matched GBM-N controls. Strikingly, many nucleotides and their biosynthetic precursors (e.g., adenosine, guanosine, and uridine monophosphate) were among the most significantly upregulated metabolites in surviving GBM-T cells (Fig. 6A). Pathway enrichment analysis confirmed that purine metabolism was the most prominently upregulated pathway (Fig. 6A), suggesting that the reconfiguration of glycolytic flux may serve to enhance nucleotide synthesis for supporting cell survival and proliferation.

To further define this metabolic state, we analyzed our previously published nCounter gene expression dataset [16]. Unlike traditional qPCR, the nCounter digital analyzer uses barcoded probes to directly count mRNA transcripts without amplification, enabling precise quantitative evaluation of gene expression (Fig. 6B) [35]. Consistent with our protein analyses, GBM-T cells exhibited transcriptional upregulation of *SLC2A1* (encoding GLUT1) and HK1, while several lower glycolytic enzymes, including *GAPDH*, *ENO1*, and *ALDOA*, were downregulated (Fig. 6B). Despite the marked decrease in LDHA protein abundance observed in chemoradiotherapy-treated GBM cells (Fig. 2C), the mRNA transcript expression of *LDHA* was increased (Fig. 6B). Discrepancies between mRNA and protein abundance are well-documented and can arise from post-transcriptional regulation, translational control, and protein stability or degradation [36]. Thus, changes in transcript levels do not necessarily predict protein abundance. It is also possible that increased *LDHA* transcription may represent a compensatory feedback response to reduced LDHA protein levels and diminished glycolytic output, as *LDHA* transcription is known to be influenced by metabolic fluctuations [37].

Additionally, genes involved in nucleotide metabolism and biosynthesis were also significantly enriched, supporting the notion that enhanced glucose uptake and upper glycolytic flux is coupled to nucleotide synthesis in GBM-T cells (Fig. 6B). Additionally, functional enrichment analysis of downregulated metabolites from our untargeted metabolomics analysis revealed an overall decrease in metabolic intermediates from many amino acid pathways (Supplementary Fig. 5A), suggesting increased utilization of amino acids in other biosynthetic process. This was further corroborated by our nCounter dataset, where many enzymes involved in amino acid breakdown and catabolism were upregulated (Supplementary Fig. 5B).

Glycolysis can fuel nucleotide biosynthesis through the diversion of upper glycolytic intermediates into the pentose phosphate pathway (PPP). G6P can be converted to 6-phosphogluconate and further processed into ribose-5-phosphate precursors essential for ribo- and deoxyribonucleotide synthesis. Similarly, F6P and G3P can be converted to sedoheptulose-7-phosphate, contributing to ribose-5-phosphate production (Fig. 6C). ¹³C₆-U-glucose tracing revealed significantly increased incorporation of heavy-labeled carbons into 6-phosphogluconate and sedoheptulose-7-phosphate in surviving GBM-T cells relative to GBM-N controls (Fig. 6C and Supplementary Table 1). These findings demonstrate that the enhanced glucose uptake and upper glycolytic activity of chemoradiotherapy-surviving GBM cells help to channel glycolytic intermediates toward PPP-mediated nucleotide synthesis, supporting anabolic demands during therapeutic stress and recovery.

**Fig. 7:**
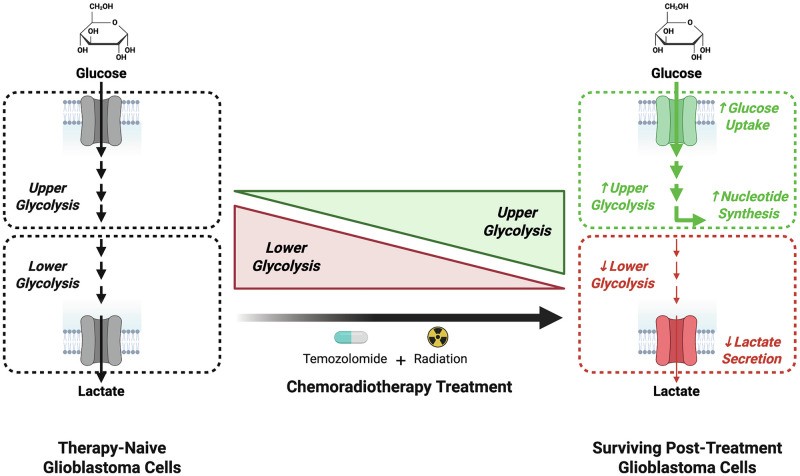
Glioblastoma cells that evade chemoradiotherapy-induced cell death exhibit a bifurcated glycolytic program. Schematic research summary depicting that chemoradiotherapy induces a coordinated reorganization of glycolytic metabolism in GBM cells. Surviving cells display increased glucose uptake and enhanced upper glycolytic activity but reduced lower glycolytic enzyme expression and lactate output. This shift promotes the diversion of glycolytic intermediates toward the biosynthetic pathways, such as nucleotide production.

Taken together, these findings establish that GBM cells surviving chemoradiotherapy exhibit a coordinated bifurcated glucose metabolic program that is conserved across diverse patient-derived in vitro and in vivo models. This metabolic state enhances glucose uptake and upper glycolytic activity while simultaneously suppressing lower glycolytic flux to divert glycolytic intermediates toward the PPP and support nucleotide biosynthesis (Fig. 7).

## Discussion

While previous studies have explored the metabolic dependencies of heterogeneous GBM populations and the adaptations that occur in response to high-dose chemo-monotherapy or radiation treatment alone [38–43], the standard-of-care for GBM involves a combination of concurrent chemotherapy and radiation [2–4]. Yet, the molecular metabolic features of tumor cells that are able to survive this combination chemoradiotherapy treatment and ultimately drive relapse remain poorly understood. Here, we employ a clinically relevant concurrent chemoradiotherapy treatment regimen in ten genetically diverse patient-derived GBM cells as well as pre-clinical PDOX animal models, which allows us to study the temporal dynamics of glucose metabolism in GBM cells that survive treatment and initiate tumor relapse.

Rather than adopting a purely “glycolytic” or “mitochondrial” phenotype, we found that GBM tumor cells that survive chemoradiotherapy exhibit enhanced glucose uptake while simultaneously suppressing downstream glycolytic activity. This coordinated glycolytic uncoupling redirects upper glycolytic intermediates toward anabolic pathways supporting nucleotide biosynthesis. In parallel, chemoradiotherapy-surviving GBM cells display markedly reduced lactate fermentation, instead channeling glycolytic end-products toward mitochondrial metabolism.

A key insight from this work is that suppression of lower glycolysis following chemoradiotherapy is not merely a passive outcome of metabolic stress but represents an active reorganization of carbon flux. By diverting intermediates away from pyruvate and lactate production, therapy-surviving cells enhance the availability of upstream carbon substrates for anabolic pathways, most notably the PPP. Isotopologue tracing experiments revealed increased labeling of 6-phosphogluconate and sedoheptulose-7-phosphate in therapy-surviving GBM cells, indicating elevated PPP flux. This rerouting coincided with increased nucleotide precursor pools and strong transcriptional enrichment of genes involved in nucleotide biosynthesis. These findings align with previous work highlighting the importance of purine and pyrimidine metabolism in GBM cells in response to genotoxic stress [43, 44]. Together, these data support a model in which surviving GBM cells progressively reorganize glycolysis to prioritize anabolic and biosynthetic demands over catabolic energy production.

Our findings complement and extend previous observations of therapy-induced mitochondrial reprogramming in GBM. We recently demonstrated that chemoradiotherapy exposure increases dependency on the MPC, channeling pyruvate toward mitochondrial metabolism and shaping the epigenetic landscape [16]. Here, we show that reduced lower glycolytic activity and lactate production are coupled with increased abundance of mitochondrial TCA cycle enzymes. These results converge on a model in which therapy-surviving GBM cells undergo a coordinated metabolic bifurcation: one arm redirects carbon flux toward biosynthesis via the PPP, while the other supports mitochondrial metabolic activity to sustain metabolic flexibility and epigenetic control. This dual program may represent a fundamental survival state that enables GBM cells to survive therapy-induced stress and reinitiate tumor formation.

## Materials and methods

Sources for all models, chemicals, reagents, antibodies, and software used in this study are presented in Supplementary Table 2.

### Isolation of patient-derived glioblastoma tumor cells

The patient-derived GBM tumor cells BT935, BT954, BT594, BT602, BT698, BT428, MBT06, and MBT222 were kindly provided by Dr. Sheila Singh (Centre for Discovery in Cancer Research, McMaster University; MTA no. MTO20-120). Dr. Singh and colleagues performed the following isolation and characterization procedures of patient-derived samples as previously described [45]. Human GBM samples were obtained from consenting patients, as approved by the Hamilton Health Sciences/McMaster Health Sciences Research Ethics Board. Upon surgical removal, tumor tissues were dissociated in PBS containing 0.2 Wünsch unit/mL Liberase (Roche) and incubated at 37 °C in a shaker for 15 min. The dissociated tissue was filtered through a 70 µm cell strainer and collected by centrifugation (450 g, 3 min). Red blood cells were lysed using ammonium chloride solution (STEMcell Technologies). The cells were washed with PBS and resuspended in NeuroCult NS-A Proliferation Medium (STEMcell Technologies) supplemented with 20 ng/mL EGF (STEMcell Technologies), 10 ng/mL bFGF (STEMcell Technologies), 2 µg/mL of Heparin (STEMcell Technologies) and 1X antibiotic-antimycotic (Anti-Anti; Gibco). The cells were then plated on ultra-low attachment plates (Corning) and propagated as tumorspheres. Additional patient-derived GBM cell lines, GBM4 (MGG4) and GBM8 (MGG8), were provided as a kind gift from Dr. Hiroaki Wakimoto (Massachusetts General Hospital, Boston, MA, USA).

While p53 status is not available for all patient-derived GBM lines used, the primary mechanistic studies were performed in BT935 cells, which harbor E286K and P72R p53 mutations. Importantly, one of the additional validation lines (BT594) was confirmed to be p53 wild-type, indicating that the metabolic adaptations we observed are conserved across patient models and occur independently of p53 status.

No commonly misidentified cell lines were used in this study, and the cell lines were authenticated by STR profiling (ATCC). All cell lines tested negative for Mycoplasma contamination using the MycoAlert® Mycoplasma Detection Kit (Lonza).

### Cell culture and treatments

Patient-derived GBM cells were maintained in serum-free DMEM/F12 medium (Gibco) supplemented with 1X N2 supplement, 1X SM1 supplement, 20 ng/mL EGF, 10 ng/mL bFGF, 2 µg/mL of Heparin, and 1X antibiotic-antimycotic and propagated as non-adherent tumorspheres.

For in vitro chemoradiotherapy treatment, patient-derived GBM cells received 25 µM of TMZ, and controls received equal volumes of DMSO vehicle. Immediately following TMZ treatment, cells were exposed to 1 Gy of X-ray irradiation using the Rad Source 2000 (Georgia, USA) and incubated at 37 °C in a 5% CO_2_ incubator for 1 h. To mimic the exposure time of chemotherapy received by patients, where the half-life of TMZ is approximately 1–2 h in circulation, the drug treatment was washed out of the patient-derived GBM cells after 1 h and replaced with fresh media. The concurrent chemoradiotherapy treatment regimen was repeated daily for 5 consecutive days. After the 5th treatment day, patient-derived GBM cells received a 2-day treatment holiday prior to collection and subsequent analysis.

### Dead cell removal

Living cells were isolated using the Dead Cell Removal Kit from Miltenyi Biotec according to the manufacturer’s protocol. Cells were pelleted by centrifugation at 600 x *g* for 5 min, and pellets were washed in 1 mL of 1X Binding Buffer. Cells were counted by trypan blue exclusion and resuspended in 100 µL of Dead Cell Removal MicroBeads per 10⁷ total cells and incubated for 15 min at room temperature. Following incubation, samples were loaded onto the LS MACS^®^ Column in a MACS magnetic separator and flow through containing living cells was collected.

### Cell count

To monitor cell viability, the trypan blue exclusion assay was used to determine the viability of cells in suspension. Trypan blue is a dye that stains dead cells due to their breached membrane integrity, allowing for the discrimination of non-viable cells. Cells were collected before and after dead-cell removal and stained 1:1 with trypan blue, and cells were counted using a Bio-Rad TC20 Cell Counter.

### 2-NBDG glucose uptake assay

Cells were plated in glucose-free DMEM media (ThermoFisher) with 150 µg/mL of 2-NBDG (ThermoFisher) for 3 h. Media was removed, and the cells were washed in PBS and then lysed in 100 µL of ice-cold lysis buffer containing 0.1M potassium phosphate and 1% (v/v) Triton X-100 adjusted to pH 10 to release 2-NBDG, and the lysate was plated in 96-well Lumox plates (Sarstedt). The amount of 2-NBDG taken up by patient-derived GBM cells in each well was quantified using a fluorometric plate reader (BioTek Synergy 2) using an excitation wavelength of 468 nm and an emission wavelength of 540 nm. A portion of the lysate (10 µL) was removed and analyzed for total protein content using the Micro BCA assay kit, and the fluorescent intensity was normalized to total cell number.

### U-^13^C_6_-glucose stable isotope tracing central carbon metabolomics

1 × 10^7^ cells were pelleted by centrifugation at 600 x *g* for 5 min. Supernatants were removed, and cells were washed in PBS. Pellets were resuspended in glucose-free DMEM media supplemented with 1X N2 supplement, 1X SM1 supplement, 20 ng/mL EGF, 10 ng/mL bFGF, 2 µg/mL of Heparin, 1X antibiotic-antimycotic, and 10 mM of [U-^13^C_6_]-glucose (Cambridge isotopes). Samples were incubated for 3 h at 37 °C in 5% CO_2_. Following incubation, cells were pelleted by centrifugation at 600 x *g* for 5 min. Supernatants were removed, and cells were washed in PBS. Polar metabolites were extracted by adding 300 µL of ice-cold methanol to the pellets, and samples were stored at –80 °C until processing.

Mass spectrometry processing of samples was performed by The Metabolomics Innovation Centre (TMIC) Victoria Node at the UVic-Genome BC Proteomics Centre, Victoria, Canada. Samples were thawed, centrifuged, and 100 μL of the supernatant was dried under nitrogen gas. In the sample residues, 50 μL of 100-mM 3-NPH solution and 50 μL of 100-mM EDC-3% pyridine solution were added. The mixtures were reacted at 30 °C for 40 min. After reaction, 200 μL of water was added to each solution. 10 μL aliquots of the resultant solutions were injected into a C18 column (2.1 × 100 mm, 1.8 µm) to quantitate carboxylic acids by UPLC-MRM/MS with (–) ion detection on an Agilent (California, USA) 1290 UHPLC coupled to an Agilent 6495 C QQQ MS instrument, according to the procedure and LC-MS parameters described previously [46]. For other metabolites, 50 μL of 20% methanol was added to dissolve the residue of each sample. 10 μL of each resultant sample solution was injected into a C18 LC column (2.1 × 100 mm, 2.5 µm) for UPLC-MRM/MS runs with (–) ion detection on a Waters (Massachusetts, USA) Acquity UPLC system coupled to a Sciex (Ontario, Canada) 6500 Plus MS instrument, with the use of a tributylamine acetate buffer–acetonitrile as the mobile phase for gradient elution (5% to 50% B over 22 min) at 0.25 mL/min and 60 °C.

### Untargeted metabolomics

Mass spectrometry metabolomics analysis was performed by The Metabolomics Innovation Centre (TMIC) Victoria Node at the UVic-Genome BC Proteomics Centre, Victoria, Canada.

A Dionex (California, USA) Ultimate 3000 UHPLC system coupled to a Thermo Scientific (Massachusetts, USA) LTQ-Orbitrap Velos Pro mass spectrometer equipped with an electrospray ionization (ESI) source was used for analysis.

Human cell samples in 300 μL 80% Methanol/water were taken out of a –80 °C freezer and placed on ice. Two 3 mm Stainless steel beads were added with 40 μL of water. Samples were vortexed for 15 s and placed on a Mixer Mill homogenizer for 30 s at 20 Hz, placed on ice for 1 min and homogenized again for an additional 30 s. 360 μL of methanol and 200 μL chloroform was added. The tube was vortex mixed for 15 s at 3000 rpm, homogenized as above for 30 s, then sonicated in an ice-water bath for 5 min and centrifuged at 21,000 × *g* (10 °C) for 10 min.

A 750-μL aliquot of the clear supernatant was taken out and transferred to a new Eppendorf safe-lock tube and stored at –80 °C. The remaining cell pellet debris was speed vacuum dried and used for protein quantitation to normalize samples. An exact microliter amount of the supernatant corresponding to 41.6 μg protein was transferred to an LC injection micro-vial and dried down under a gentle nitrogen gas flow in a nitrogen evaporator at 35 °C. The residue was dissolved in 50 μL of 1:1 Methanol/Chloroform. Five or seven microlitres were injected to run reversed-phase LC-MS for detection and relative quantitation of polar to lipophilic metabolites.

A microliter aliquot corresponding to 41.6 μg protein of the clear supernatant was precisely taken out and transferred to another 1.5-mL Eppendorf safe-lock tube and dried down under nitrogen gas flow in a nitrogen evaporator at 35 °C. 400 μL of 60% methanol/water was added to rehydrate, followed by 30 s vortex and 1 min sonication. After the addition of 180 μL of chloroform to the tube, vortex mixing for 20 s followed by centrifugal clarification at 21,000 x *g* (10 °C) for 10 min to separate the whole phase into two distinct layers. 360 μL of the upper aqueous phase was carefully transferred to an LC injection micro-vial and dried down in a nitrogen evaporator at 35 °C. The residue was reconstituted in 100 μL of 70% acetonitrile. After a further 1:1 dilution with 70% Acetonitrile, five or seven microlitres were injected and run HILIC-MS for detection and relative quantitation of very polar and hydrophilic metabolites.

Reversed-phase (RP)-UPLC-MS runs were carried out for analysis of polar to hydrophobic metabolites with the use of a Waters C8 UPLC column (2.1 × 50 mm, 1.7 μm) for chromatographic separation. The mobile phase was (A) 0.01% formic acid in water and (B) 0.01% formic acid in acetonitrile-isopropanol (1:1, v/v) for positive-ion detection. For negative-ion detection, the mobile phase was (A) 0.01% formic acid in water and (B) 0.01% formic acid in acetonitrile-isopropanol (1:1, v/v). The efficient gradient was 5% to 50% B in 5 min; 50% to 100% B in 15 min, and 100% B for 2 min before the column was equilibrated for 5 min at 5% B between injections. The column flow rate was 0.4 mL/min, and the column temperature was maintained at 55 °C. The LTQ Velo Pro Orbitrap MS instrument was operated in the survey-scan mode with full-mass and high-resolution Fourier transform (FT) MS detection at a mass resolution of 60,000 FWHM @ m/z 200. The mass scan range was 80 to 1800 m/z for positive-ion and 80 to 1800 m/z negative-ion detection. Along with the LC-MS data acquisitions, LC-MS/MS data were also acquired for a pool of several samples using collision-induced dissociation (CID) and high-energy C-trap dissociation (HCD). For LC-MS/MS, the 5 most abundant ions from each survey scan were chosen for subsequent CID and HCD in each duty cycle, with the normalized collision energies of 25% to 40%.

For analysis of very polar metabolites, Hydrophilic interaction chromatography (HILIC) –MS using a Waters Amide column (2.1 × 100 mm, 1.7 µm) was performed, with positive-ion or negative-ion detection in each round of two LC injections per sample.

For HILIC-MS, the mobile phase was (A) 0.01% formic acid in water and (B) 0.01% formic acid in acetonitrile. The efficient gradient was 95% for 1.5 min; 95% to 10% B in 11 min; 10% B for 2.5 min before the column was equilibrated at 95% B for 5 min between injections. The column flow rate was 0.3 mL/min, and the column temperature was maintained at 40 °C. The MS instrument was operated in the survey-scan mode with full-mass FTMS detection at a mass resolution of 60,000 FWHM @ m/z 200. The mass scan range was m/z 50 to 1000 for both positive-ion and negative-ion detection. Along with the LC-MS data acquisitions, LC-MS/MS data were also acquired for a few samples using collision-induced dissociation (CID) and high-energy C-trap dissociation (HCD). For LC-MS/MS runs, the 5 most abundant ions from each survey scan were chosen for subsequent CID and HCD in each duty cycle, with the normalized collision energies at 25 to 40%.

Enrichment analysis of untargeted metabolomics data was performed using MetaboAnalyst 5.0.

### Lactate assay

Lactate measurements were performed using the Lactate Assay Kit from Sigma-Aldrich (MAK064) according to the manufacturer’s instructions. Serum-free growth media were collected and frozen at –80 °C until processing. On the day of the experiment, media samples were diluted 10X in lactate assay buffer prepared according to the manufacturer’s instructions. 5 μL of diluted media was added to a clear 96-well plate and adjusted to a final volume of 25 μL with lactate assay buffer for a total 50X dilution. 25 μL of the Master Reaction Mix containing 23 μL of lactate assay buffer, 1 μL of lactate enzyme mix and 1 μL of lactate probe was added to each well. Colorimetric absorbance was measured at 570 nm using a SpectraMax Plus Plate Reader, and values were normalized to total protein concentration.

### Protein extraction and Western immunoblotting

Patient GBM cells were collected and centrifuged at 500 x *g* for 5 min at 4 °C. Pellets were resuspended in RIPA lysis buffer (25 mM Tris pH 7.6, 150 mM NaCl, 1% NP-40, 1% sodium deoxycholate, 1% SDS) containing 1% protease inhibitor cocktail (PIC) and phosphatase inhibitors. Whole cell lysates were incubated on ice for 45 min and then sonicated for 30 s. The samples were centrifuged at 20,000 x *g* for 15 min at 4 °C, and the supernatants containing the proteins were collected. Protein concentrations were determined using the colorimetric Micro BCA assay kit (Life Technologies) according to the manufacturer’s instructions. Equal amounts of protein were boiled in Laemmli sample buffer (Bio-Rad) containing 5% β-mercaptoethanol for 5 min and then resolved by SDS-PAGE. Protein was transferred onto nitrocellulose membranes (BioRad). Membranes were coated with Ponceau S dye to detect total protein concentration for normalization. Membranes were then washed in ddH_2_O to remove Ponceau S before blocking. Membranes were blocked in 5% non-fat milk in PBST (PBS, 0.05% Tween 20) for 45 min, then washed in PBST and incubated in the appropriate primary antibody overnight at 4 °C with shaking. The primary antibodies were prepared at a 1:1000 dilution in 1% BSA in PBST. The following day, membranes were washed in PBST before incubating in appropriate horseradish peroxidase (HRP)-conjugated secondary antibodies. Secondary antibodies were prepared at a 1:10 000 dilution in 5% non-fat milk in PBS and added to the membranes for 1.5 h at room temperature. Following secondary antibody incubation, membranes were washed, and proteins were detected using Clarity ECL Western substrate (Bio-Rad) and visualized using the ChemiDoc MP imaging system (Bio-Rad) in chemiluminescent setting. Semi-quantitative analysis of the protein densitometry signal was performed using ImageJ software. Whole lane total protein normalization was performed using the Ponceau S stain from the same gel on which the respective protein of interest was probed. All western blot quantifications were performed using at least three biological replicates. Each replicate corresponds to an independent treatment batch, run on separate gels across different days. To address blot-to-blot variability, the fold-change of treated samples was normalized relative to respective control samples from the experiment, setting the control values to a baseline value of 1 [47]. This approach is a standard practice for Western blot normalization and ensures robust data interpretation. Original uncropped western blot images are source data are provided in the supplementary material.

### Animal studies

All experiments involving animals were approved by the University of Manitoba’s Animal Care Committee (protocol #21-027). Non-obese diabetic (NOD) severe combined immunodeficient (SCID) IL2R gamma null (NSG) mice (NOD.Cg-*Prkdc*^*scid*^ *Il2rg*^*tm1Wjl*^/SzJ) were acquired from the CancerCare Manitoba in-house breeding colony, courtesy of Dr. Jody Haigh. Animals were housed in IVC caging and held according to the Guidelines of the Canadian Council on Animal Care and the Animal Care and Use Policy of the University of Manitoba. Irradiated feed (5P76—Prolab® IsoPro® RMH 3000) was used, and caging and bedding were sterilized by steam autoclave. Animals had continuous access to food and water for the study duration. Room ambient temperature was 21–23 °C with a relative humidity target of 50%, but within a range of 30–60%. Light cycle was 12 h on/12 h off, beginning with lights on at 6:00 a.m.

For modeling the progression of recurrent GBM, 6 male and 6 female mice aged 7–9 weeks old received intracranial xenografts of patient-derived BT935 GBM cells. Animals were anesthetized using isoflurane gas (5% induction and 2.5% maintenance), and 1 × 10^6^ patient-derived BT935 GBM cells suspended in 5 µL of PBS were injected into the frontal lobe in a nonrandomized, nonblinded fashion. Following confirmation of successful tumor engraftment by T2 MRI imaging using an MR Solutions cryogen-free FlexiScan 7T system (MR Solutions, Guildford, Surrey, UK), equal numbers of male and female mice were randomly assigned to receive no treatment (primary tumor controls) or standard chemoradiotherapy. No blinding was done for animal studies. The chemoradiotherapy protocol was adapted to suit the physiology of NSG mice as previously described [29]. The animals receiving chemoradiotherapy were given 50 mg/kg TMZ treatment by oral gavage, followed immediately by 2 Gy of craniospinal X-ray irradiation using the Rad Source 2000 coupled with a lead body shield on the first day of treatment, followed by 50 mg/kg/day TMZ for the following 4 consecutive days. The University of Manitoba’s Animal Care Committee has no maximal tumor size/burden restrictions for orthotopic brain tumor studies. Animals were sacrificed at humane endpoints as defined by 20% reduction from peak body weight or significant clinical deterioration (i.e., evidence of pain, neurological symptoms, paralysis, etc.) as decided in consultation with the veterinarian.

### Immunohistochemistry

Following deparaffinization of xenograft tumor samples, antigen retrieval was performed in citrate buffer at 95–100 °C for 20 min. Slides were washed and treated for 10 min for endogenous peroxidase, and again washed in 1X PBS. The samples were blocked with 3% sheep serum, then incubated with primary antibodies overnight at 4 °C. Slides were washed and incubated with Biotin-conjugated secondary antibody for 2 h at room temperature. Streptavidin/HRP (Life Technologies) was then added for 30 min followed by development with DAB and counterstaining with hematoxylin. Finally, coverslips were mounted with Permount (Fisher Scientific). Images were captured using a Zeiss AxioImager in the Quantitative Imaging, Phenotyping and Sorting (QuIPS) Platform located in the Paul Albrechtsen Research Institute, CancerCare Manitoba. Images were quantified using ImageJ Fiji software (National Institutes of Health). Color was deconvoluted from IHC images, and the Hematoxylin/DAB stains were quantified at a set threshold value for each protein. The mean gray intensity value of the DAB signal was plotted for each image quantified.

### Bioinformatics analysis

Previously published RNA-sequencing data from patient-derived xenograft tumor samples from Qazi et al. [29] (10.1016/j.celrep.2022.111420) were accessed through correspondence with the authors. Gene set enrichment analysis (GSEA) was performed by running RNA-Seq data from Naïve and TMZRT-treated tumors through gene sets from the Molecular Signatures Database (mSigDB) using GSEA software (Broad Institute) [48, 49]. Our previously published nCounter® Metabolic Pathways Panel gene expression data from Martell et al. [16] (10.1093/neuonc/noaf008) was analyzed using NanoString nSolver software.

### Statistical analysis

Statistical analysis was performed in GraphPad Prism 10. Statistical parameters, including the exact value of n and the statistical significance, are reported in the figures and figure legends. No power calculations were utilized to determine the required sample size for in vitro and in vivo experiments. For in vivo studies, experiments included an equal number of mice in each treatment group with at least *n* = 6 animals (3 males and 3 females) per experimental condition; no animals were excluded from analysis. For in vitro studies, all experiments were performed on a minimum of three independent biological replicates. No sample size calculation was made for the analysis of data accessed from public repositories, as all available cases in the cited repositories were included. To assess significant differences between single measurements of two groups of normally distributed data, the unpaired two-tailed Student’s *t* test was used. To assess significant differences between more than two groups of normally distributed data, we performed one-way or two-way analysis of variance (ANOVA), followed by a Dunnett’s multiple comparisons test when each mean was only compared to the control mean or a Šídák’s multiple comparisons test when comparing a select set of means. The data are presented as mean + standard error of the mean (SEM) unless otherwise specified. Differences were considered statistically significant at *P* < 0.05.

## Supplementary information

Supplementary Information

Supplementary Tables

Uncropped Blots

## Data Availability

Raw files for untargeted metabolomics mass spectrometry data have been deposited to the Global Natural Products Molecular Networking (GNPS) repository [50] as a part of MassIVE (MSV000093877, 10.25345/C55D8NR5C). Data can be downloaded through the following FTP link: ftp://massive.ucsd.edu/MSV000093877/. This paper reports on previously published and publicly available datasets from Martell et al. [16] (10.1093/neuonc/noaf008) and Qazi et al. [29]. (GSE195681). All data generated or analyzed during this study are included in this manuscript and its supplementary information files. Original uncropped western blot images are source data are provided in the supplementary material. All other data reported in this paper will be shared by the lead contact upon request.
