# Supplementary material for: Glioblastoma cells that evade chemoradiotherapy-induced cell death exhibit a bifurcated glycolytic program
Source: Cell Death Dis. 2026 Mar 25;17(1):348. doi: 10.1038/s41419-026-08646-9 (PMC13039382; doi:10.1038/s41419-026-08646-9)
Supplement: Supplementary file 1 — Supplementary Information [file 41419_2026_8646_MOESM1_ESM.pdf]

SUPPLEMENTARY INFORMATION

Glioblastoma cells that evade chemoradiotherapy-induced cell death exhibit a bifurcated glycolytic program

Martell and Kuzmychova *et al.*

SUPPLEMENTARY FIGURES

Supplementary Figure 1

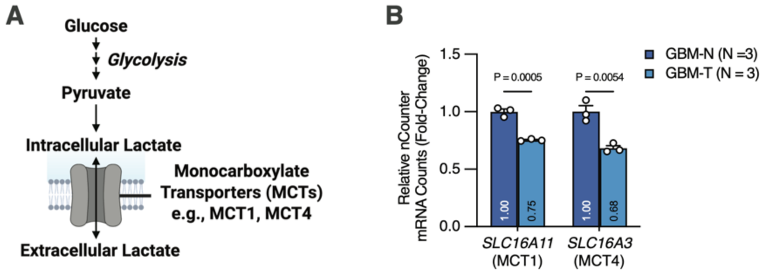

Supplementary Figure 2

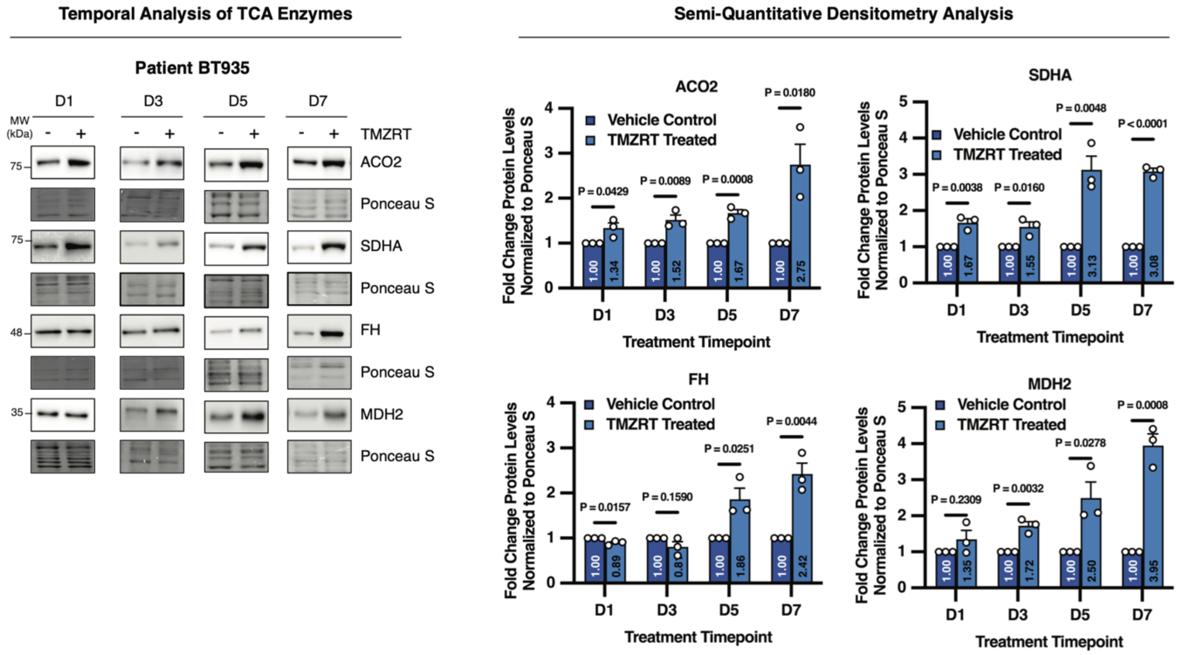

Supplementary Figure 3

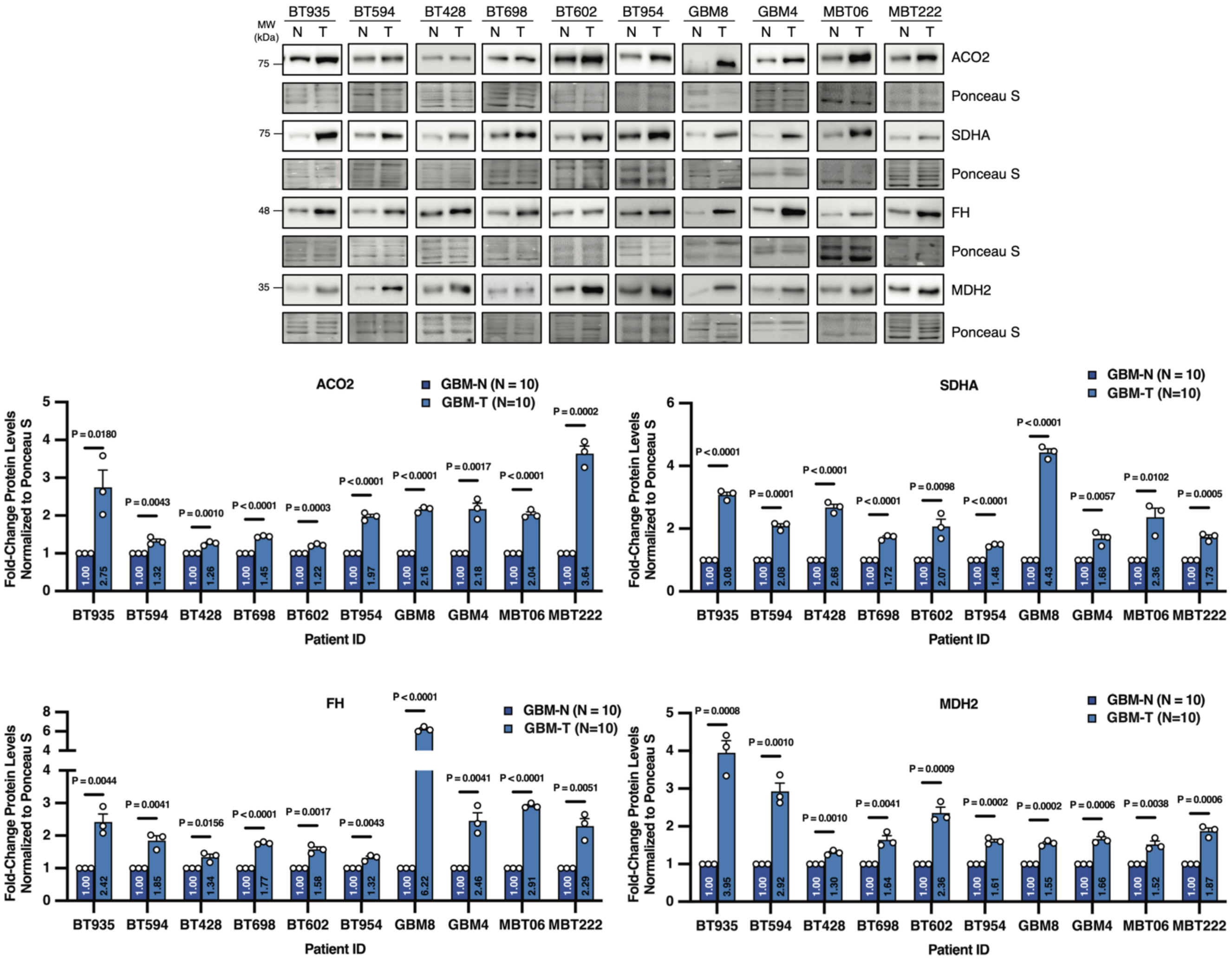

Supplementary Figure 4

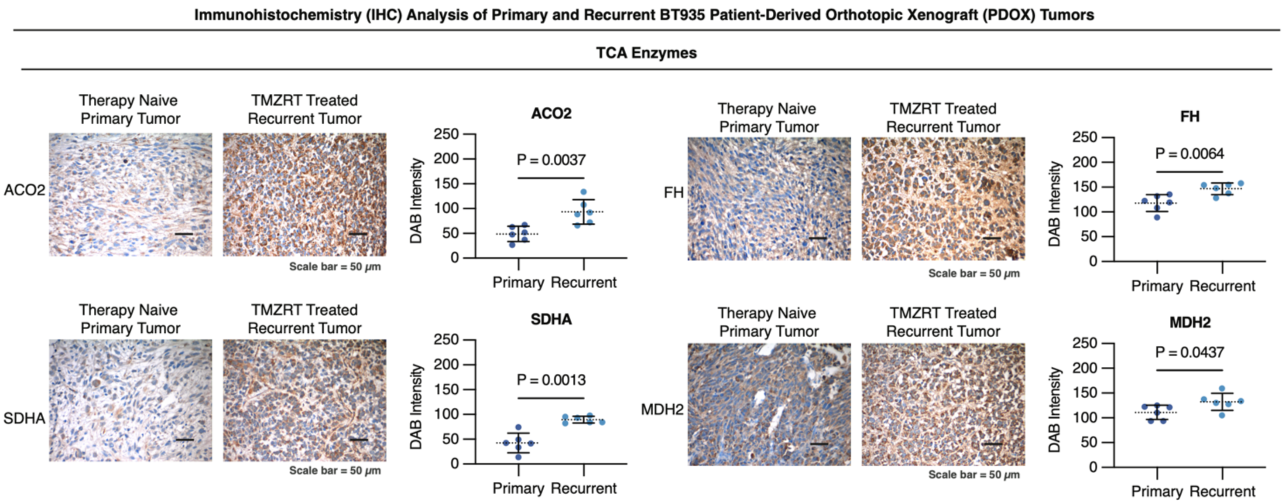

Supplementary Figure 5

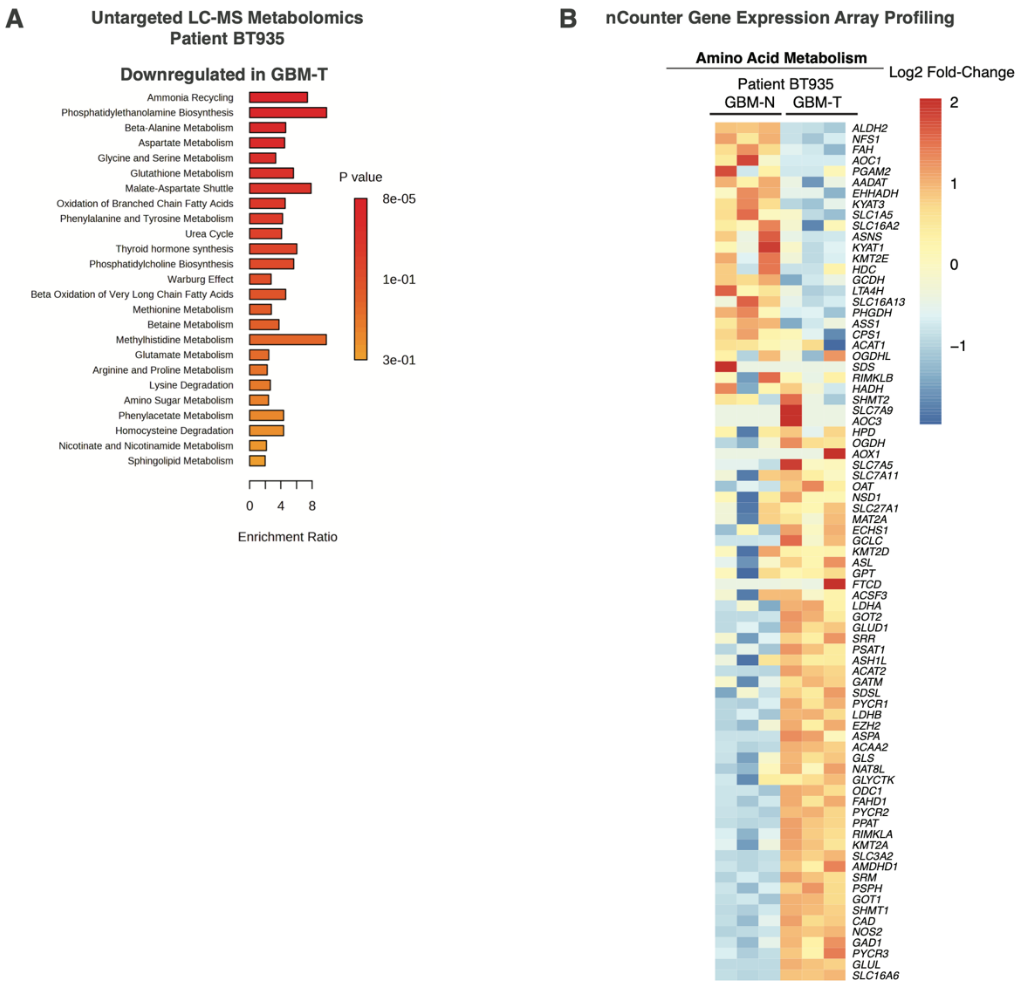

SUPPLEMENTARY FIGURE LEGENDS

Supplementary Figure 1. (A) Schematic diagram depicting lactate production and extracellular excretion. **(B)** Normalized mRNA counts of lacate monocarboxylate transporters (MCTs), MCT1 (*SLC16A1*) and MCT4 (*SLC16A3*), detected using NanoString nCounter Metabolic Pathway panel analysis in BT935-T versus BT935-N cells. N = 3 biological replicates/group, mean + SEM analyzed using unpaired two-tailed t-test.

Supplementary Figure 2. BT935 patient-derived GBM cells treated with placebo control or TMZRT chemoradiotherapy were collected at D1, D3, D5, and D7 time-points and living cells were isolated and subjected to immunoblot and semi-quantitative densitometry analysis of protein levels for TCA enzymes ACO2, SDHA, FH, MDH2. N = 3 biological replicates/group, mean + SEM analyzed using unpaired two-tailed t-test

Supplementary Figure 3. N=10 matched therapy-naïve and post-treatment patient-derived GBM cells were subjected to immunoblot analysis and semi-quantitative densitometry analysis of protein levels for TCA enzymes ACO2, SDHA, FH, MDH2. N=3 biological replicates per sample where graphs represent mean + SEM, analyzed using unpaired two-tailed t-test.

Supplementary Figure 4. Representative micrographs and quantifications of DAB intensity from IHC analysis for TCA enzymes ACO2, SDHA, FH, MDH2, in therapy naïve primary versus TMZRT-treated recurrent PDOX BT935 GBM tumor tissues. N = 6 samples/group analyzed using unpaired two-tailed t-test.

Supplementary Figure 5. (A) Bar graph of the functional enrichment of metabolic pathways for metabolites downregulated in BT935-T versus BT935-N cells based on untargeted metabolomics analysis. **(B)** NanoString nCounter Metabolic Pathway panel analysis and heatmap depicting log2 fold-change expression of amino acid metabolism genes in BT935-T versus BT935-N cells from N = 3 biological replicates/group.
