# Supplementary material for: Glioblastoma cells that evade chemoradiotherapy-induced cell death exhibit a bifurcated glycolytic program
Source: Cell Death Dis. 2026 Mar 25;17(1):348. doi: 10.1038/s41419-026-08646-9 (PMC13039382; doi:10.1038/s41419-026-08646-9)

UNCROPPED BLOTS

Figure 2B

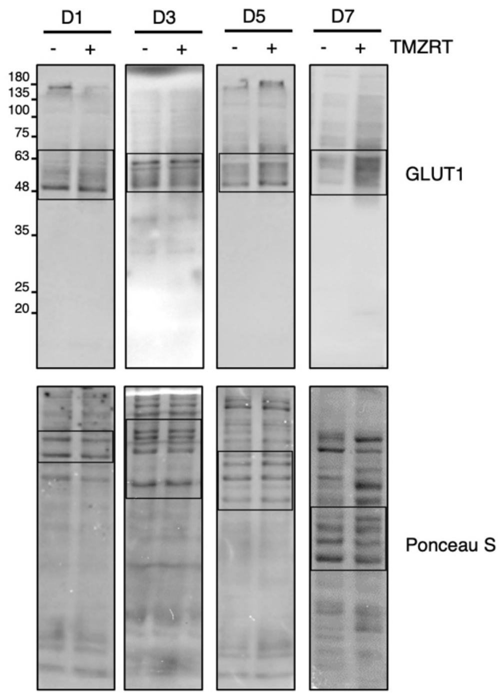

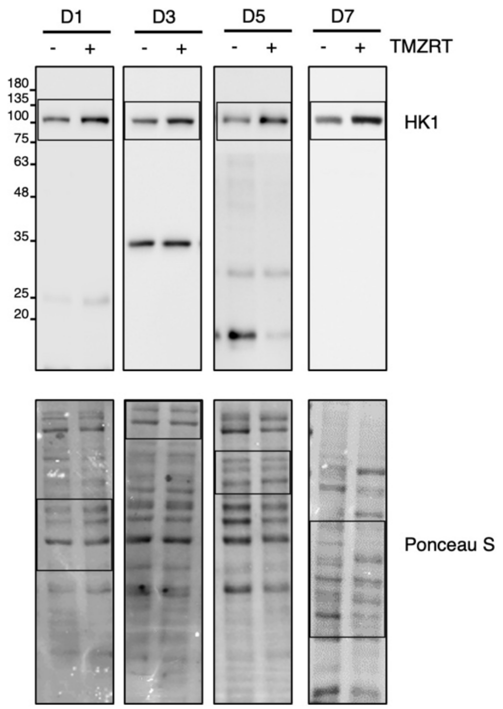

UNCROPPED BLOTS

Figure 2B

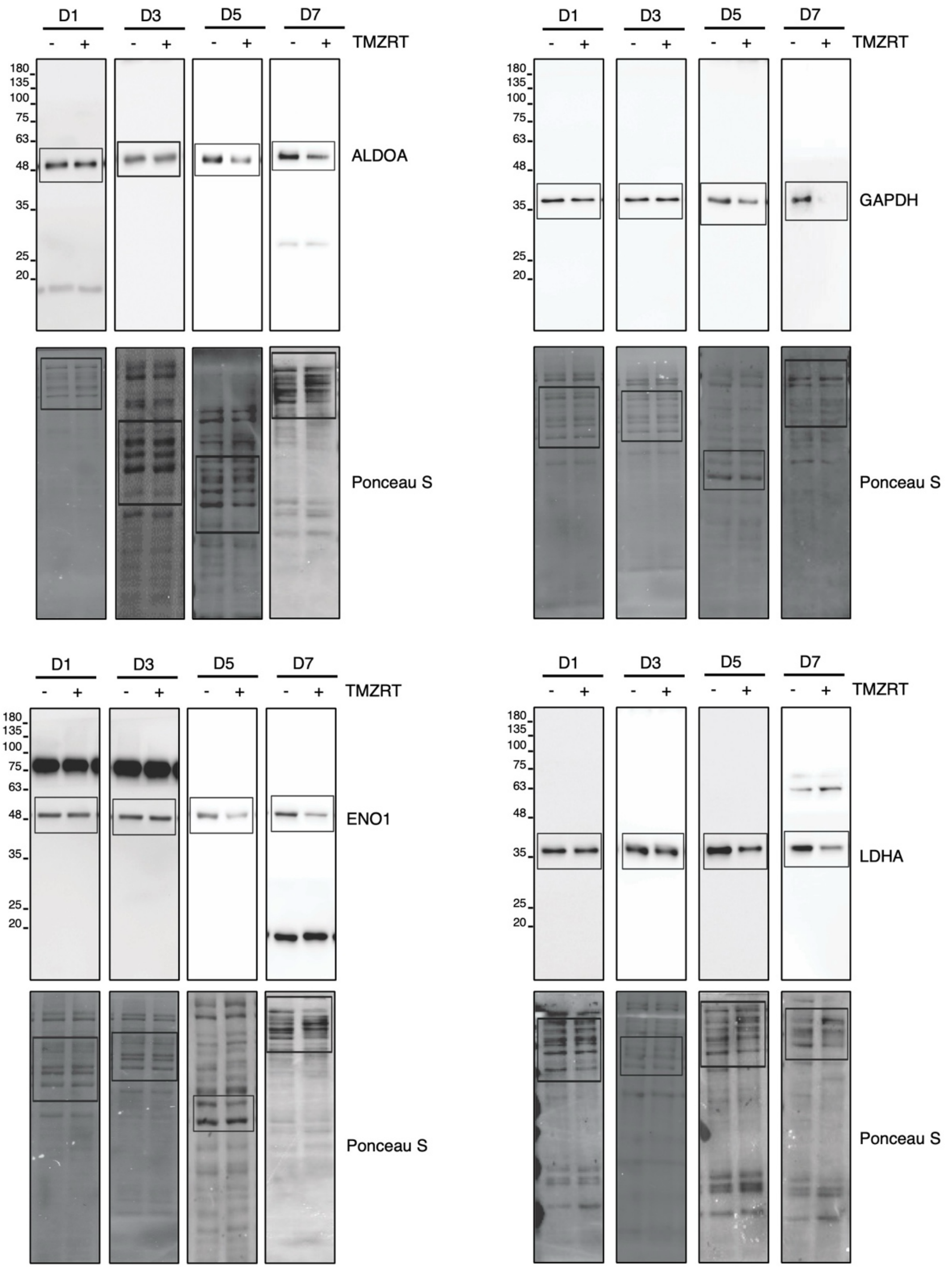

UNCROPPED BLOTS

Figure 4C

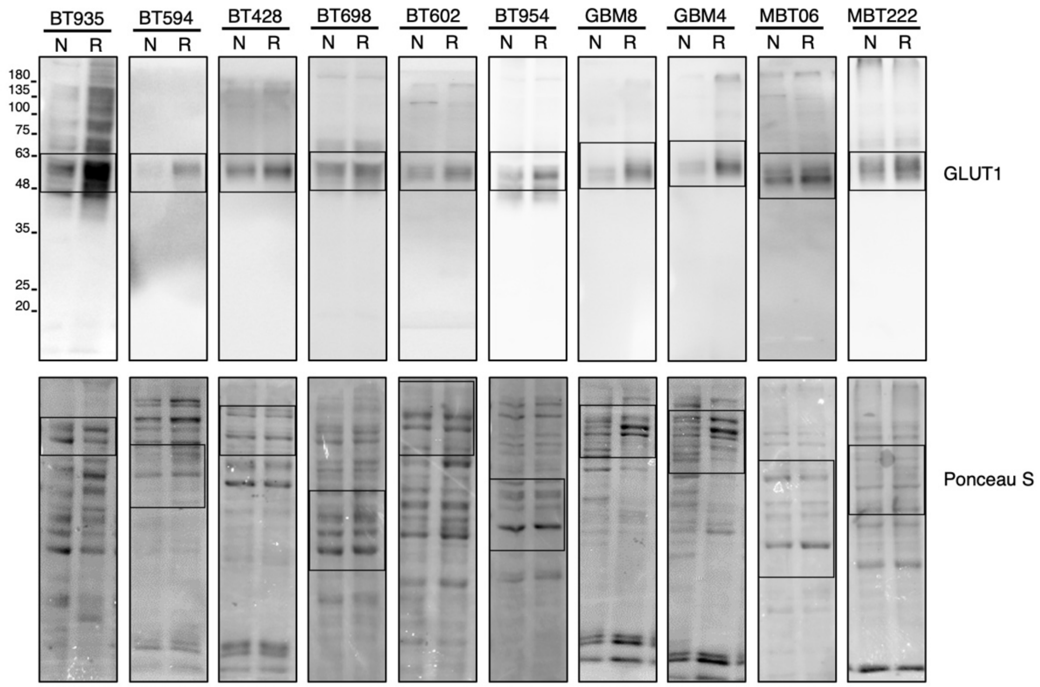

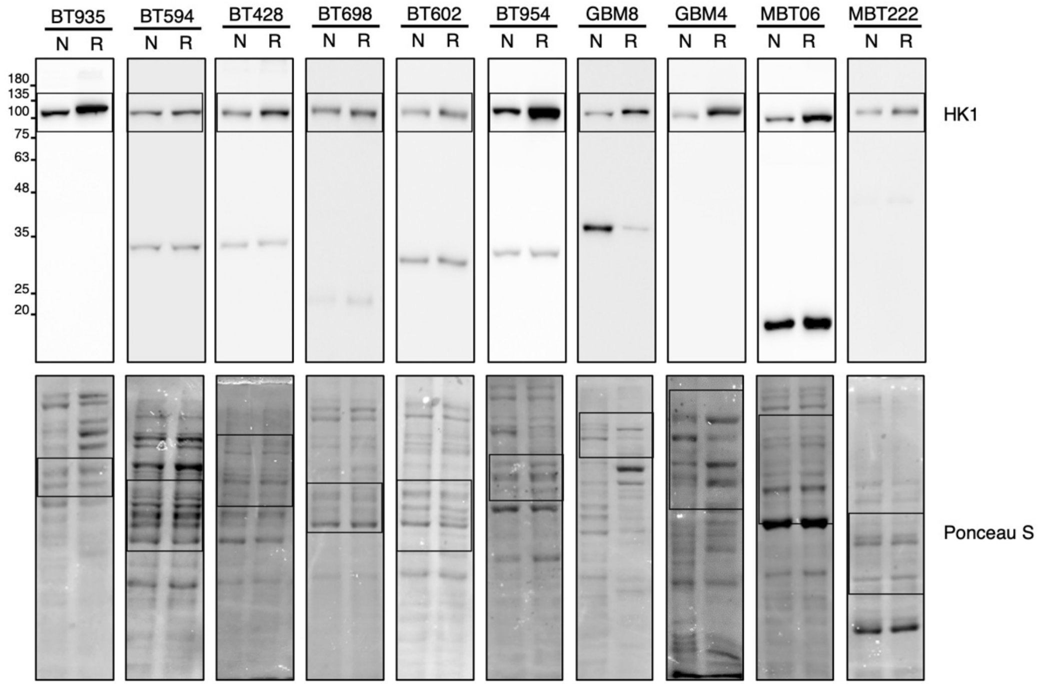

UNCROPPED BLOTS

Figure 4C

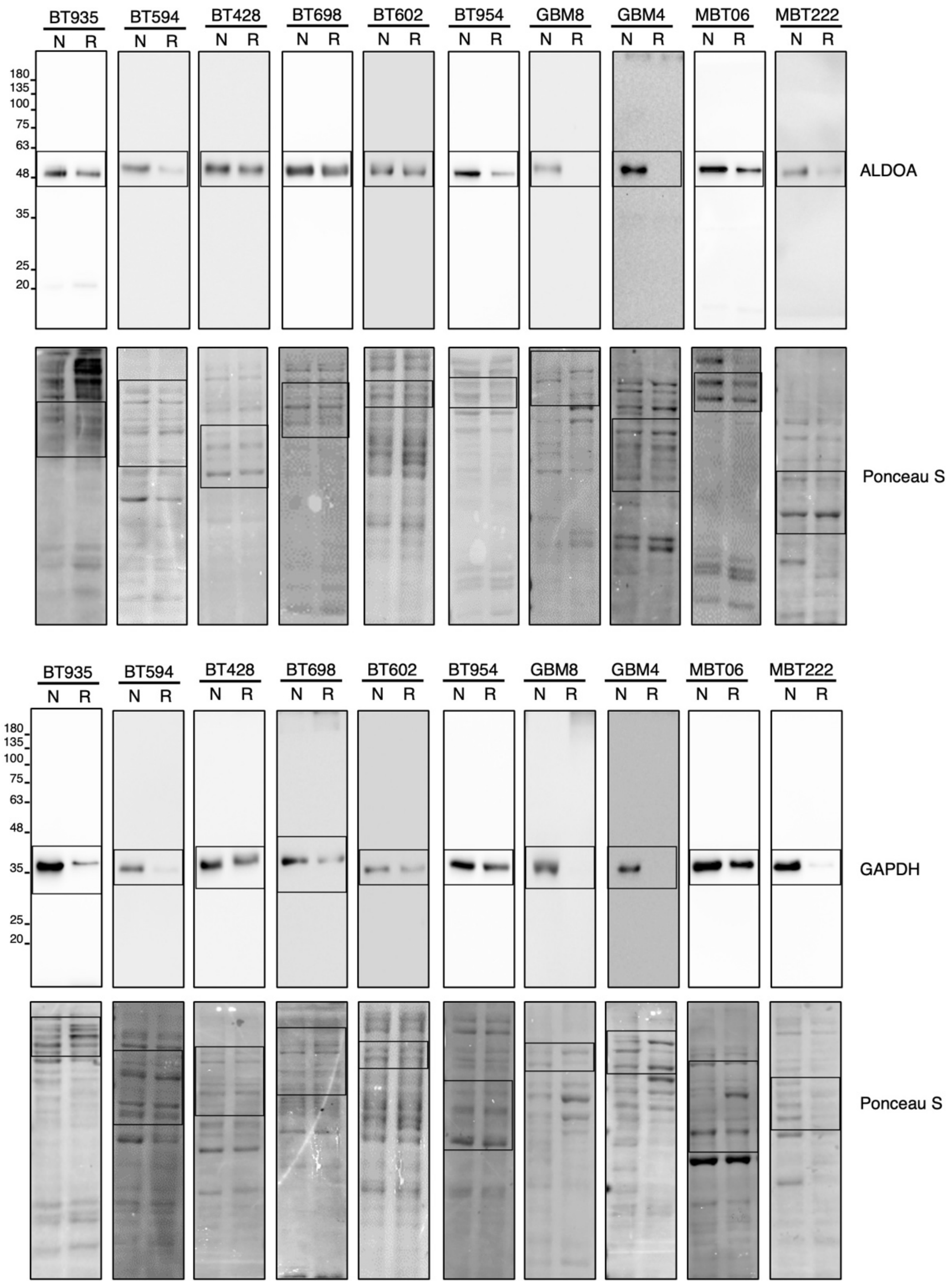

UNCROPPED BLOTS

Figure 4C

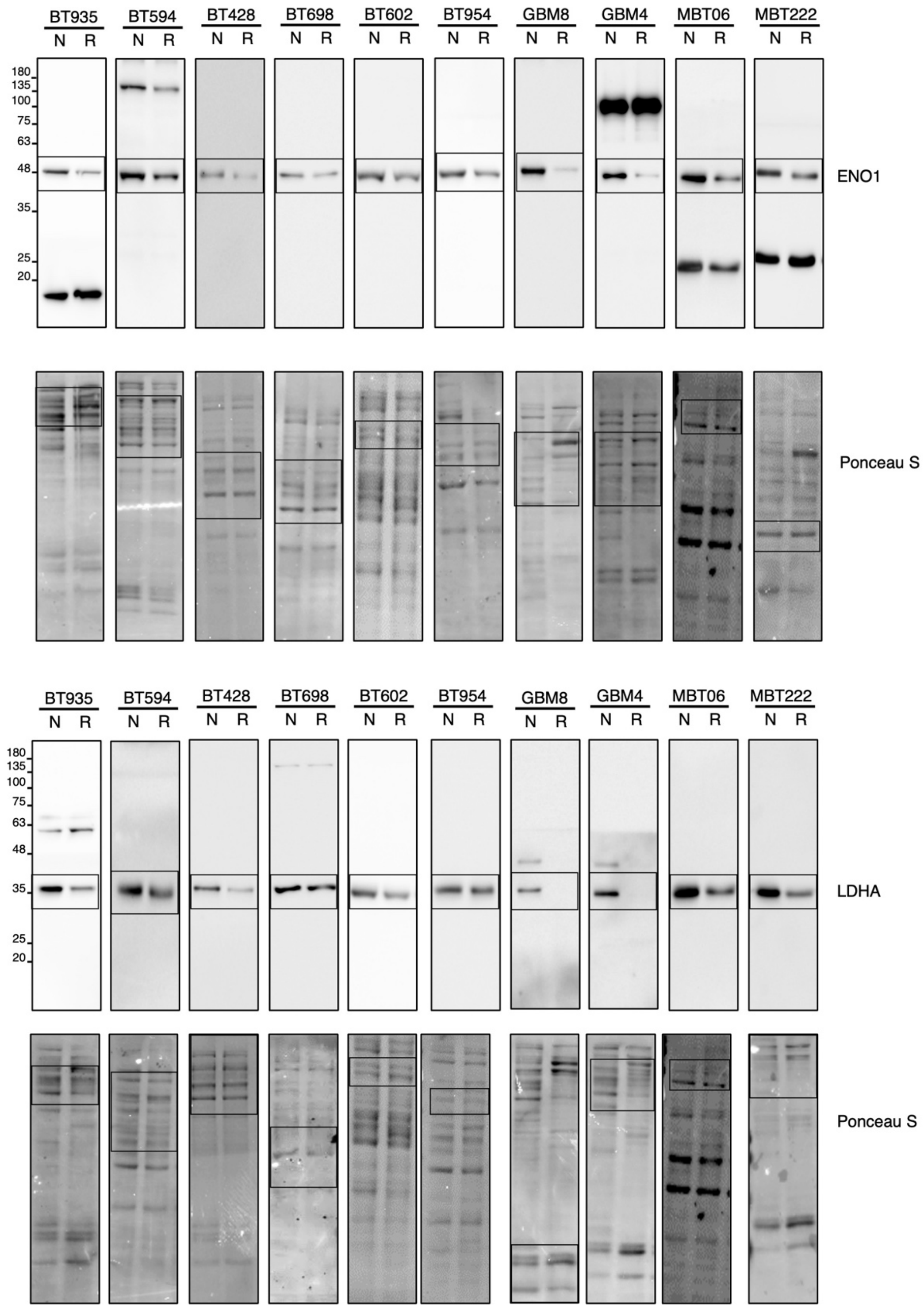

UNCROPPED BLOTS

Supplementary Figure 2

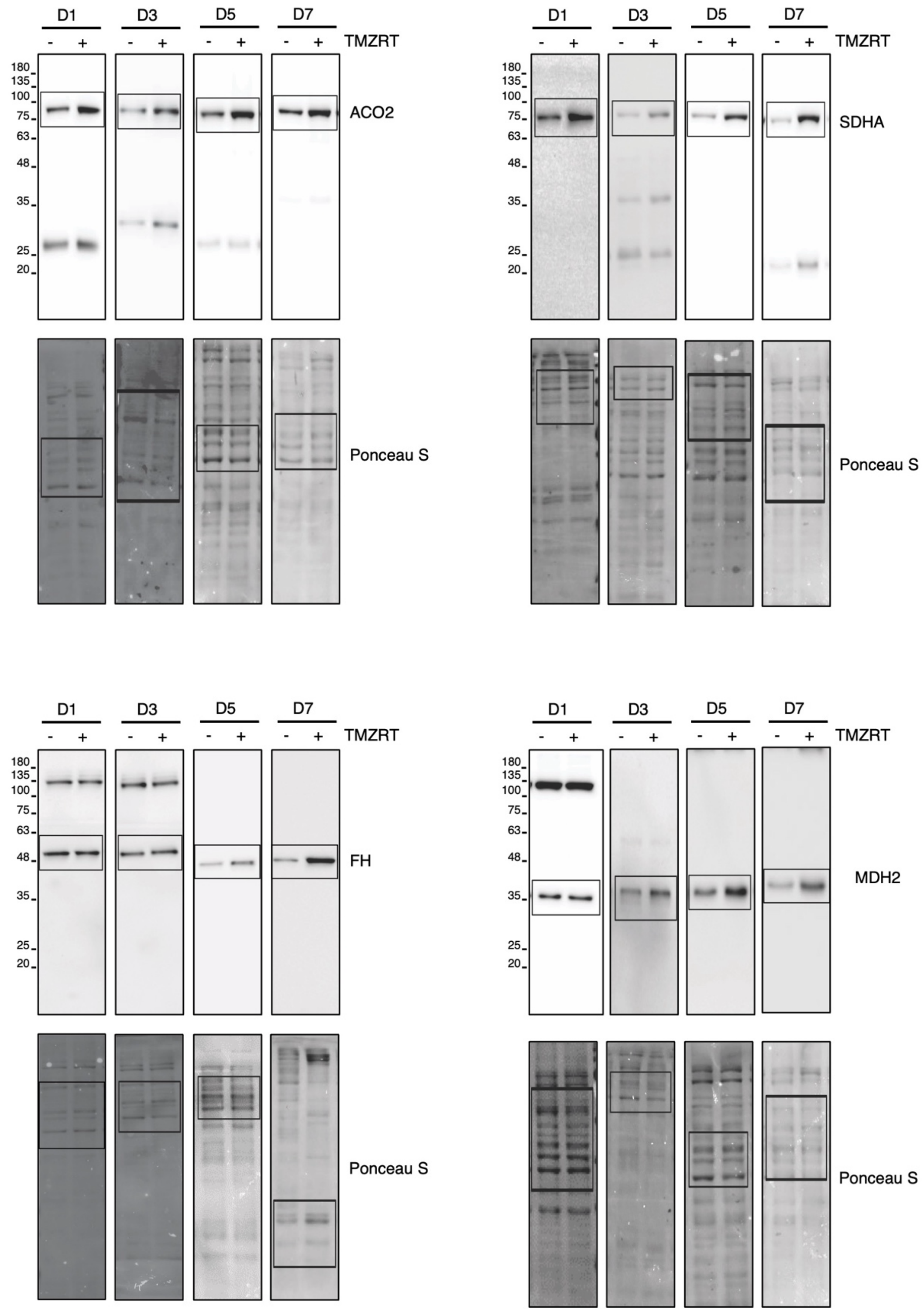

UNCROPPED BLOTS

Supplementary Figure 3

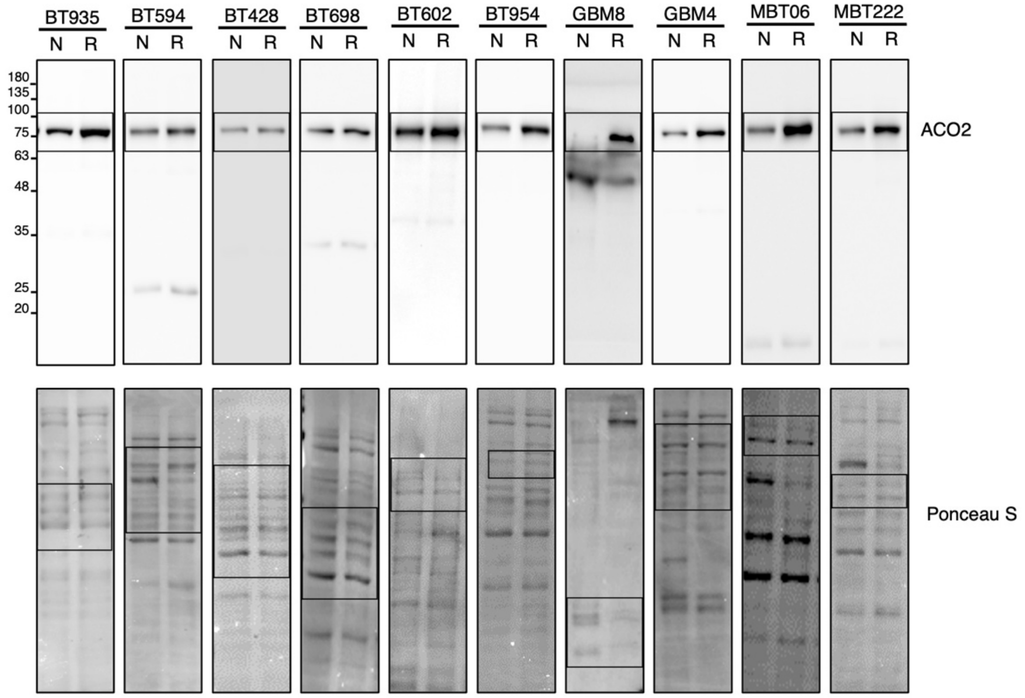

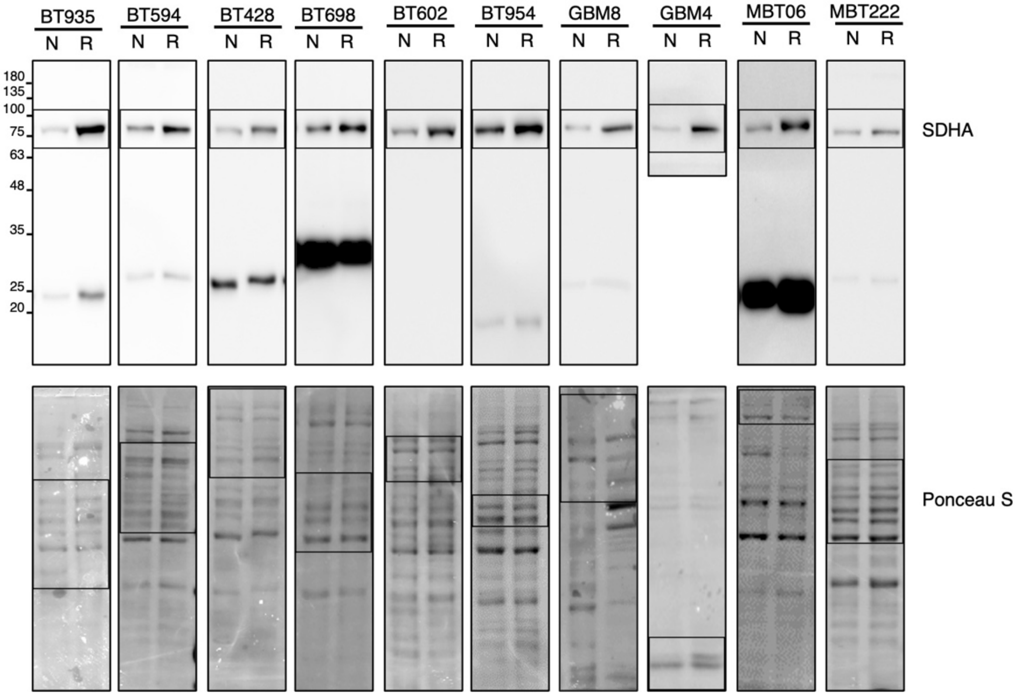

UNCROPPED BLOTS

Supplementary Figure 3

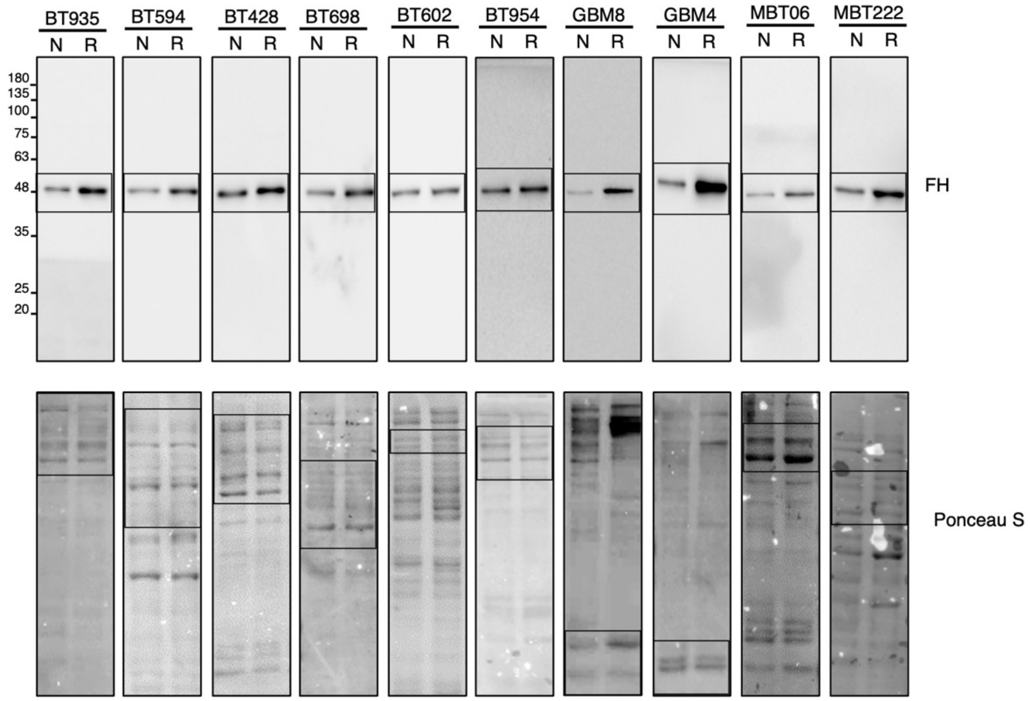

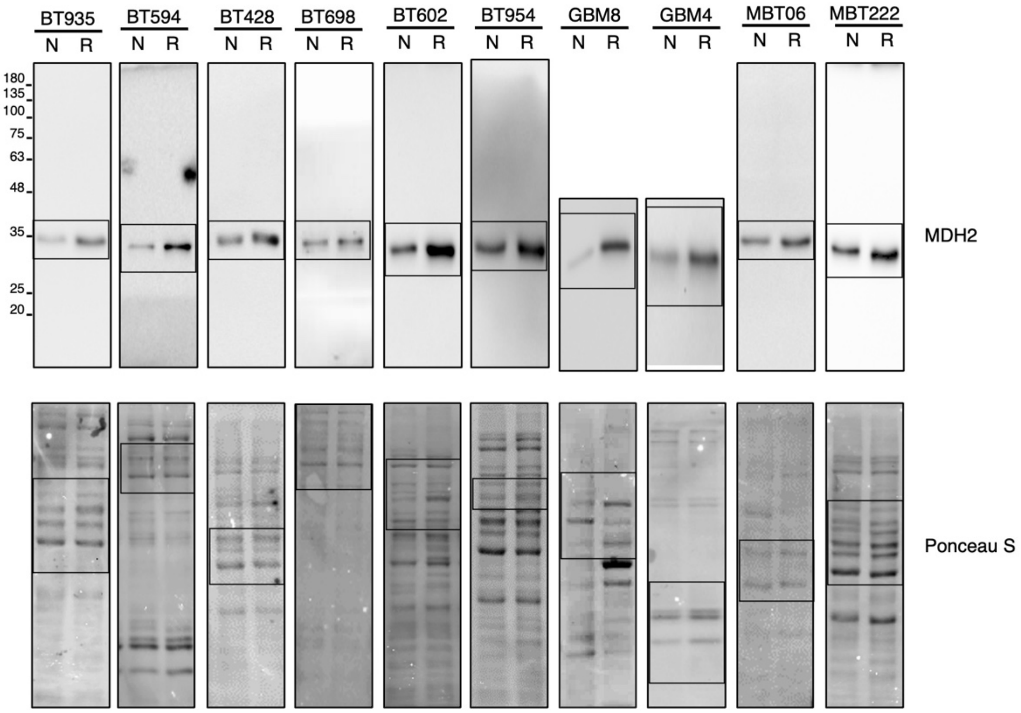

Supplement: Supplementary file 3 — Uncropped Blots [file 41419_2026_8646_MOESM3_ESM.pdf]
